# Midbrain Dopamine Neurons Defined by TrpV1 Modulate Psychomotor Behavior

**DOI:** 10.3389/fncir.2021.726893

**Published:** 2021-11-11

**Authors:** Gian Pietro Serra, Adriane Guillaumin, Sylvie Dumas, Bianca Vlcek, Åsa Wallén-Mackenzie

**Affiliations:** ^1^Unit of Comparative Physiology, Department of Organism Biology, Uppsala University, Uppsala, Sweden; ^2^Oramacell, Paris, France

**Keywords:** glutamate, VGLUT2, VMAT2, co-release, transient receptor vanilloid, amphetamine, sensitization, psychostimulant

## Abstract

Dopamine (DA) neurons of the ventral tegmental area (VTA) continue to gain attention as far more heterogeneous than previously realized. Within the medial aspect of the VTA, the unexpected presence of TrpV1 mRNA has been identified. TrpV1 encodes the Transient Receptor Potential cation channel subfamily V member 1, TRPV1, also known as the capsaicin receptor, well recognized for its role in heat and pain processing by peripheral neurons. In contrast, the brain distribution of TrpV1 has been debated. Here, we hypothesized that the TrpV1^+^ identity defines a distinct subpopulation of VTA DA neurons. To explore these brain TrpV1^+^ neurons, histological analyses and Cre-driven mouse genetics were employed. TrpV1 mRNA was most strongly detected at the perinatal stage forming a band of scattered neurons throughout the medial VTA, reaching into the posterior hypothalamus. Within the VTA, the majority of TrpV1 co-localized with both Tyrosine hydroxylase (Th) and Vesicular monoamine transporter 2 (Vmat2), confirming a DA phenotype. However, TrpV1 also co-localized substantially with Vesicular glutamate transporter 2 (Vglut2), representing the capacity for glutamate (GLU) release. These TrpV1^+^/Th^+^/Vglut2^+^/Vmat2^+^ neurons thus constitute a molecularly and anatomically distinct subpopulation of DA-GLU co-releasing neurons. To assess behavioral impact, a *TrpV1*^*Cre*^-driven strategy targeting the *Vmat2* gene in mice was implemented. This manipulation was sufficient to alter psychomotor behavior induced by amphetamine. The acute effect of the drug was accentuated above control levels, suggesting super-sensitivity in the drug-na ve state resembling a “pre-sensitized” phenotype. However, no progressive increase with repeated injections was observed. This study identifies a distinct TrpV1^+^ VTA subpopulation as a critical modulatory component in responsiveness to amphetamine. Moreover, expression of the gene encoding TRPV1 in selected VTA neurons opens up for new possibilities in pharmacological intervention of this heterogeneous, but clinically important, brain area.

## Introduction

Dopamine (DA) neurons of the ventral tegmental area (VTA) are critical to limbic and cognitive functions, and, hence, exert a major impact on behavioral regulation. Consequently, their dysfunction is correlated with the severe neuropsychiatric disorder, including addiction, attention deficit/hyperactivity disorder (ADHD), schizophrenia, and the affective/cognitive non-motor domain of Parkinson’s disease (PD) (Björklund and Dunnett, 2007). Major efforts are, therefore, aimed at elucidating the neurobiological underpinnings of the VTA in order to advance prediction, prevention, and treatment prospects of brain dysfunction implicating this brain area.

The VTA was long considered a homogeneous DA structure. However, it is today well recognized that VTA DA neurons coexist with glutamate (GLU)- and GABA-signaling neurons, and that DA, GLU, and GABA neurons show regional distribution within the VTA, also reflected in their projections (Lammel et al., 2011; Hnasko et al., 2012; Watabe-Uchida et al., 2012; Beier et al., 2015; Menegas et al., 2015). In addition, several layers of heterogeneity have been reported within the VTA DA system, including electrophysiological properties, afferent/efferent projections, responsiveness to positive and negative reinforcers, the ability for neurotransmitter co-release, gene expression, and vulnerability to disease (Roeper, 2013; Brichta and Greengard, 2014; Pupe and Wallén-Mackenzie, 2015; Morales and Margolis, 2017; Poulin et al., 2020). The VTA can be further subdivided into anatomically distinct subnuclei based on the distribution of DA neurons. For example, medial VTA subnuclei consist of the interfascicular nucleus (IF) and rostral linear nucleus (RLi), whereas more lateral VTA subnuclei include the paranigral (PN) and parabrachial pigmented (PBP) nuclei. In addition, the caudal linear nucleus (CLi) is sometimes, but not always, grouped with the VTA, and there is also a medial-lateral distribution within each VTA subnucleus. VTA DA neurons send extensive forebrain projections, primarily to the nucleus accumbens and cerebral cortex. The *Th* and *Slc18a2* genes encoding the Tyrosine hydroxylase (TH) and Vesicular monoamine transporter 2 (VMAT2) proteins that are essential to DA production (TH) and vesicular packaging (VMAT2), respectively, are, by necessity, expressed in all neurons defined as dopaminergic, while other genes important to DA cell function may vary more. For example, the *Slc6a3 (Dat)* gene, encoding the dopamine transporter (DAT), which mediates DA reuptake after vesicular release, shows a medial^*low*^-lateral^*high*^ expression pattern in the VTA (Lammel et al., 2008; Papathanou et al., 2018). Furthermore, the density of DA neurons differs across VTA subnuclei (Morales and Margolis, 2017).

The medial VTA is of particular interest for several reasons. The mesoaccumbal DA projection, which is strongly associated with reward processing, motivation, and behavioral reinforcement, shows a topographical projection pattern. Medial VTA DA neurons innervate the medial aspect of the nucleus accumbens shell (mAcbSh), and lateral VTA DA neurons target the lateral shell and core areas within the nucleus accumbens (Ikemoto, 2007; Poulin et al., 2018). DA release in the mAcbSh is associated with the reinforcing effects of both natural and drug rewards, and drugs of abuse increase DA levels more efficiently in the mAcbSh than lateral shell and core (Di Chiara and Imperato, 1988; Pontieri et al., 1995; Lüscher, 2016). Furthermore, medial VTA DA neurons show higher expression of the *Vglut2* gene than DA neurons of the lateral VTA (Kawano et al., 2006; Li et al., 2013; Papathanou et al., 2018). The presence of the VGLUT2 protein allows GLU packaging into presynaptic vesicles for fast excitatory neurotransmission (Hnasko and Edwards, 2012). Within the VTA, the *Vglut2* gene is expressed in GLU neurons and DA-GLU co-releasing neurons, the latter primarily, but not uniquely, located in medial subareas (reviewed in Trudeau et al., 2014; Trudeau and El Mestikawy, 2018; Eskenazi et al., 2021). DA-GLU neurons project within the nucleus accumbens to mAcbSh, where they primarily target cholinergic interneurons and medium spiny neurons (Kawano et al., 2006; Li et al., 2013; Chuhma et al., 2014; Mingote et al., 2015; Poulin et al., 2018). Conditional knockout (cKO) of *Vglut2* in DA neurons in mice (*Vglut2^*lx/lx*^; Slc6a3^*Cre/wt*^*) results in modest deficits in emotional behavior and vigor (Birgner et al., 2010; Fortin et al., 2012; Wang et al., 2017) but strongly alters the behavioral response to psychostimulants (Birgner et al., 2010; Hnasko et al., 2010; Alsiö et al., 2011; Fortin et al., 2012; Papathanou et al., 2018). Together, the results of several cKO studies converge in a model where GLU co-release plays an intricate role in reward responsiveness and behavioral reinforcement mediated by DA (Eskenazi et al., 2021). However, knowledge is still sparse, and improved resolution in dissecting out the role of this complex neurotransmitter phenotype should be useful to increase current understanding of the VTA.

In addition to the mixture of different neurotransmitter phenotypes in the VTA, also molecularly defined subtypes of DA neurons have recently been identified. These provide yet another level of heterogeneity and complexity but also render distinct DA neurons taggable and distinguishable from each other. These molecular fingerprints have been derived from gene expression studies using microarray (Chung et al., 2005; Greene et al., 2005; Viereckel et al., 2016) and, more recently, transcriptomics methodology (Poulin et al., 2014; La Manno et al., 2016; Tiklová et al., 2019; Phillips et al., 2021). These studies are often based around the quest to identify molecular properties that distinguish VTA DA neurons from those in substantia nigra *pars compacta* (SNc), and that might help explain their different vulnerability to PD. However, genes that might provide information of VTA DA neurons in other conditions, such as neuropsychiatric disorder, have also been identified. One such example is *NeuroD6*, which has consistently been reported to show higher expression levels in the VTA compared to the SNc in most microarray and transcriptomics-based studies. Anatomical mapping has revealed NeuroD6 to define a modest DA subtype regionally distributed in the VTA (Khan et al., 2017; Kramer et al., 2018; Bimpisidis et al., 2019). Functional assays showed that NeuroD6 is critical to DA cell survival and vulnerability to PD (Khan et al., 2017; Kramer et al., 2018) while recent behavior analysis has identified a role for NeuroD6-positive VTA DA neurons in approach behavior and psychostimulant response (Bimpisidis et al., 2019).

Another gene, which has been identified in a microarray approach as higher expressed in the VTA than SNc, is *TrpV1* (Viereckel et al., 2016). This gene encodes the transient receptor potential cation channel subfamily V member 1 (TRPV1; also known as the capsaicin receptor) (Caterina et al., 1997), a nonselective cation channel highly expressed in certain sensory neurons, but for which the brain distribution has been elusive and, hence, the topic of debate (Cavanaugh et al., 2011; Ramírez-Barrantes et al., 2016). However, using reporter gene expression driven by a *TrpV1^*Cre/*+^* mouse transgene to allow detection with high sensitivity and precision, *TrpV1* expression could be distinctly detected in a contiguous band of neurons reaching from the caudal hypothalamus through to the VTA, including the medially located subnucleus IF (Cavanaugh et al., 2011). While described already 10 years ago, this finding has remained largely unexplored. However, a subsequent report not only identified TrpV1 mRNA within the medial VTA but also showed that TrpV1^+^ VTA neurons were positive for both Th and Vglut2, suggesting a DA-GLU neurotransmitter phenotype. The same study demonstrated GLU release in the AcbSh upon optogenetic stimulation in *TrpV1*^*Cre/wt*^ mice, thus identifying a mesoaccumbal projection of combined TrpV1/GLU identity (Viereckel et al., 2016).

Given the recent demonstration of the medial distribution of TrpV1 mRNA within the VTA, its co-expression with both DA and GLU markers, and GLU release in the *TrpV1*^*Cre*^ mesoaccumbal projection, we hypothesized that TrpV1 is a molecular marker for a distinct subpopulation of VTA neurons of DA-GLU identity. This was tested by addressing the distribution pattern and molecular fingerprint of TrpV1^+^ neurons in mice. Furthermore, to determine if TrpV1^+^ VTA neurons contribute to behavioral regulation, a new cKO mouse line was generated in which the prerequisite for vesicular DA packaging, VMAT2, was abrogated in *TrpV1*^*Cre*^ neurons. Control and cKO mice were analyzed in behavioral paradigms relevant to the VTA, including psychomotor response upon amphetamine challenge.

## Results

### TrpV1-Positive Cells Are Primarily Present in the Medial VTA and Excluded From SNc

**FIGURE 1:**
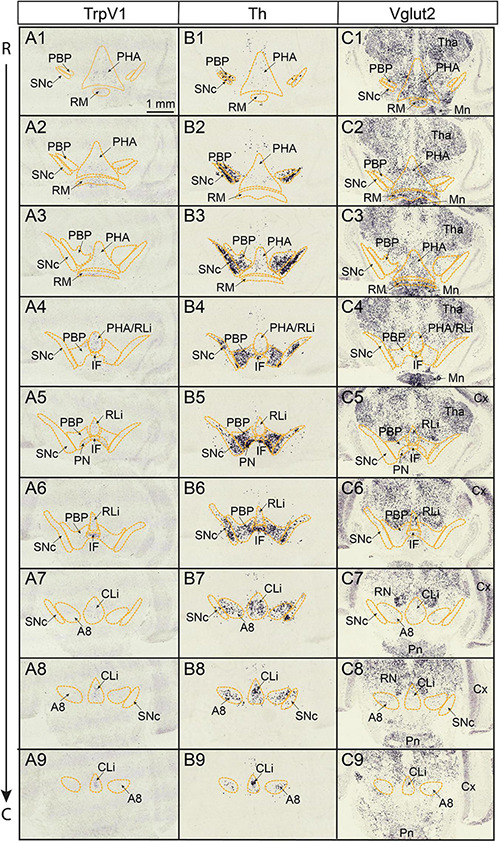
TrpV1 mRNA is detected in scattered medial cells in a continuous manner reaching from the posterior hypothalamus rostrally throughout the VTA caudally. TrpV1, Th, Vglut2 mRNAs analyzed by colorimetric *in situ* hybridization (CISH) in serial coronal brain sections at postnatal day (P) 3. The rostral end of series at the levels of PHA, RM, and PBP; the caudal end of series at the level of CLi, A8. **(A1–A9)** TrpV1; **(B1–B9)** Th; **(C1–C9)** Vglut2. Scale bar; 1 mm. A8, A8 dopamine area; CLi, caudal linear nucleus; Cx, cerebral cortex; IF, interfascicular nucleus; Mn, mammillary nucleus; PBP parabrachial pigmented nucleus; PHA posterior hypothalamic nucleus; RLi, rostral linear nucleus; Pn, pontine nucleus; PN, paranigral nucleus; RM, retromammillary nucleus; RN, red nucleus; SNc, substantia nigra *pars compacta*; VTA, ventral tegmental area; Tha, thalamus; R, rostral; C, caudal.

Based on the original report identifying TrpV1-positive (TrpV1^+^) neurons primarily in the border area connecting the ventro-medial midbrain and caudal hypothalamus, with the strongest expression in the IF subnucleus (Cavanaugh et al., 2011), it was of interest to study *TrpV1* gene expression in this brain region in more detail. Colorimetric *in situ* hybridization (CISH) was performed on serial sections from newborn mice of postnatal day 3 (P3). TrpV1 mRNA (Figures 1A1–A9) was analyzed with Th (Figures 1B1–B9) and Vglut2 (Figures 1C1–C9) mRNAs as references for DA and GLU neurons throughout this brain region. The P3 stage was selected based on our previous microarray analysis, which identified elevated *TrpV1* gene expression levels in VTA compared to SNc at this postnatal stage (Viereckel et al., 2016). Here, scattered, but distinct, TrpV1^+^ cells were detected in a continuum stretching rostrally from the posterior hypothalamic nucleus (PHA) and retromammillary nucleus (RM) of the caudal hypothalamus through to, and including, all VTA subnuclei (IF, RLi, CLi, PN, and PBP), as well as the A8 DA area of the caudal midbrain (Figures 1A1-A9 and Supplementary Figure 1). The density of TrpV1^+^ neurons in the VTA was visibly higher in medial (IF, RLi, CLi, and medial PN) than lateral (lateral PN, PBP) VTA subnuclei (Figures 1A1–A9). Furthermore, the lateral DA cell group of the SNc was devoid of TrpV1 mRNA (Figures 1A6,A7).

### TrpV1^+^ VTA Neurons Are Mainly of Mixed DA-GLU Identity (TrpV1^+^/Th^+^/Vglut2^+^)

**FIGURE 2:**
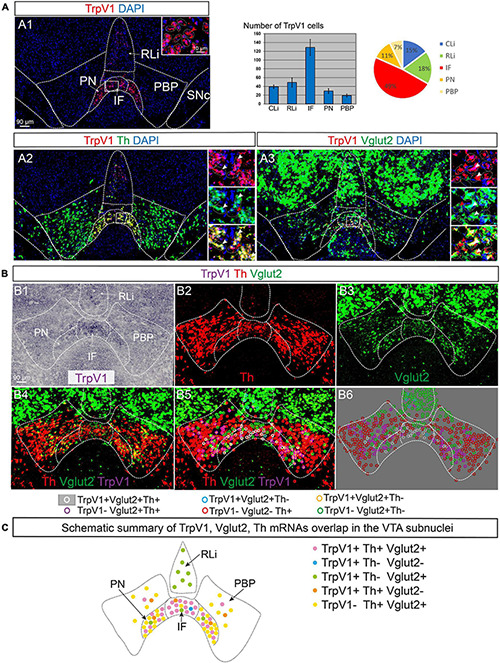
TrpV1 mRNA primarily in medial VTA subnuclei co-localizes strongly with Th and Vglut2 mRNAs. TrpV1, Th, and Vglut2 mRNAs were analyzed by fluorescent and colorimetric *in situ* hybridization (FISH and CISH) in serial coronal brain sections at postnatal day (P) 3. DAPI is used for the detection of cell nuclei. **(A1–A3)** FISH: TrpV1 (**A1**; a table and a pie chart show result of counting of TrpV1-positive cells per VTA subnucleus, *N* = 3 mice, serial sections throughout the VTA, allowing for analysis of adjacent sections); TrpV1/Th **(A2)**; TrpV1/Vglut2 **(A3)**; closeup’s in insets; cells indicated by dotted lines. The yellow color indicates colocalization of red and green fluorophores. FISH-positive cells are indicated in insets by arrowheads; white arrows indicate co-labeling of red and green fluorophores. **(B)** Triple labeling by combined CISH/FISH visualized in separate channels **(B1–B3)** and merged **(B4)**: TrpV1 (**B1**, CISH), Th (**B2**, FISH), Vglut2 (**B3**, FISH), merged TrpV1/Th/Vglut2 **(B4)**; colored circles indicate double and triple mRNA labeling (**B5** histological section; **B6**-colored circles indicating positive cells in B5 superimposed on gray background for clarity; **B5,B6** legends indicate mRNA detected per colored circle). **(C)** A schematic summary of results in **(A,B)**, an illustrated map of TrpV1/Vglut2/Th overlap in VTA subnuclei. Scale bars, 90 μm; 10 μm (insets). See Table 1 for cell counting. IF, interfasicular nucleus; PN, paranigral nucleus; PBP, parabrachial pigmented nucleus; RLi, rostral linear nucleus; VTA, ventral tegmental area.

To define neurotransmitter identity and molecular properties of the neuronal population in the VTA identified as positive for TrpV1 mRNA, a battery of histological analyses was performed. Using fluorescent *in situ* hybridization (FISH), the neurotransmitter identity of TrpV1^+^ neurons was first addressed (Figure 2). Co-localization analysis at P3 was performed assessing Th, Vglut2, and the vesicular inhibitory amino acid transporter (Viaat) mRNAs to enable detection of DA, GLU, and GABA neurons, respectively. Similar to CISH, FISH analysis detected TrpV1 mRNA in the VTA, primarily in the IF, followed by RLi, CLi, PN, and PBP (Figure 2A1). Counting of cells positive for TrpV1 mRNA showed that 49% were located in the IF subnucleus, while the corresponding percentage in other VTA subnuclei was lower (18% in RLi; 15% CLi; 11% PN; and 7% PBP) (Figure 2A1).

TrpV1 co-localized substantially with Th mRNA. More than 90% of TrpV1^+^ cells were positive for Th in the IF, PN, and PBP (Figure 2A2 and Table 1). These are all VTA subnuclei defined by a strong DA (Th^+^) phenotype. In contrast, RLi contains fewer DA (Th^+^) neurons, and, here, less TrpV1/Th co-labeling was observed (less than 5%). NoTrpV1/Th co-localization was observed in the PHA and RM, an expected finding as these hypothalamic areas are largely devoid of Th mRNA and, instead, are glutamatergic (Supplementary Figure 1).

**TABLE 1:** TrpV1mRNA and its extent of co-localization with a range of neurotransmitter and neuronal subtype markers.

**Characterisation of Trpv1 mRNA-positive phenotype in VTA subnuclei**					

	**IF**	**PN**	**PBP**	**RLi**	**CLi**
%Trpv1 **Th^+^**	95%	92%	95%	3%	73%
%Trpv1 **Vmat2^+^**	75%	77%	90%	2%	80%
%Trpv1 **Vglut2^+^**	89%	82%	60%	100%	33%
%Trpv1 **Viaat^+^**	3%	0	0	31%	9%
%Trpv1 **Dat^+^**	0	14%	40%	0	0
%Trpv1 **NeuroD6^+^**	6%	8%	20%	0	5%
%Trpv1 **Grp^+^**	14%	34%	23%	0	0
%Trpv1 **(Th^+^ Vglut2^–^)**	9%	18%	38%	0%	68%
%Trpv1 **(Vglut2^+^ Th^–^)**	5%	3%	3%	97%	27%
%Trpv1 **(Vglut2^–^ Th^–^)**	1%	0%	0%	0%	0%
%Trpv1 **(Vglut2^+^ Th^+^)**	85%	79%	59%	3%	5%
% (Vglut2 Th) **Trpv1^+^**	85%	30%	15%	almost no Th/Vglut2 coloc	

*Result of counting of double- or triple-positive cells obtained in *in situ* hybridization analysis at postnatal day 3 (P3). *N* = 3 mice per detection, serial sections throughout the VTA. Percentage of co-labeling between TrpV1 and one or two other mRNAs (in bold). Example, 95% of TrpV1-positive cells in IF are positive for Th mRNA.*

Addressing a GLU neurotransmitter phenotype, TrpV1/Vglut2 co-labeling analysis showed that the far majority of TrpV1^+^ cells throughout the VTA-hypothalamus (PHA, RM) continuum were positive for Vglut2 (Figure 2A3, Table 1, and Supplementary Figure 1). However, a substantially lower degree of overlap was observed between TrpV1 and Vglut2 in the CLi (33%) than in the RLi (100%) and IF (89%). This was due to the variable amount of both TrpV1 and Vglut2 mRNAs in different VTA subnuclei (Table 1). In contrast to the substantial TrpV1/Vglut2 co-labeling throughout the VTA and hypothalamus, few, if any, TrpV1^+^ cells were positive for Viaat mRNA, indicating a lack of a GABA phenotype. TrpV1 co-labeling with Viaat was only observed in the RLi, the area with low co-labeling with Th (Supplementary Figure 1 and Table 1).

Triple-detection, achieved by combining CISH (TrpV1) and FISH (Th and Vglut2) detection methods, was performed to allow assessment of single and dual neurotransmitter phenotypes (DA *vs.* GLU *vs.* DA-GLU) (Figures 2B1–B6 and Table 1). This analysis revealed that very few TrpV1^+^ neurons in the VTA were positive for either Th or Vglut2 mRNAs (Figures 2B4–B6,C and Table 1). TrpV1^+^/Th^+^/Vglut2^–^ (DA) neurons were primarily present in PBP while TrpV1^+^/Th^–^/Vglut2^+^ (GLU) neurons represented the most common TrpV1^+^ phenotype in RLi, in a continuum with PHA and RM (Supplementary Figure 1). However, the far majority of TrpV1^+^ neurons were positive for both Th and Vglut2 mRNAs (TrpV1^+^/Th^+^/Vglut2^+^), thus demonstrating the histological properties of a DA-GLU phenotype (Figures 2B4–B6,C and Table 1). For example, in the IF, where most TrpV1^+^ neurons reside, 85% of TrpV1^+^ cells were positive for both Th and Vglut2 (TrpV1^+^/Th^+^/Vglut2^+^) (Table 1).

While the majority of TrpV1^+^ neurons showed a DA-GLU identity, not all DA-GLU neurons were positive for TrpV1 (Table 1). In the IF subarea, containing the largest proportion of TrpV1^+^ cells, 85% of Th^+^/Vglut2^+^ (DA-GLU) neurons were positive for TrpV1 (TrpV1^+^/Th^+^/Vglut2^+^). More modest numbers were obtained for the other VTA subnuclei (PN, 30%; PBP, 15%; barely at all in RLi and CLi) (Table 1). The results suggest that TrpV1 defines a subpopulation of DA-GLU VTA neurons, which show a medial^*high*^–lateral^*low*^ distribution within VTA subnuclei IF, PN, and PBP (Figure 2C).

To pinpoint the temporal expression of *TrpV1*, serial brain sections derived from embryonic day 14.5 (E14.5), P12, and adult (9 weeks) mice were prepared to complement the analysis at P3. Also, here, TrpV1, Th, and Vglut2 mRNAs were analyzed on adjacent sections. TrpV1 mRNA was detected within the developing VTA at E14.5 but was very low at P12 and in adulthood (Supplementary Figure 2). A similar profile was evident for the hypothalamic nuclei (Supplementary Figure 2). Taken together, histological analyses identify a perinatal peak of TrpV1 mRNA in the medial aspect of the VTA/PHA/RM region between E14.5 and P3, and its subsequent downregulation.

### TrpV1 Co-localizes Substantially With Vmat2 but Not With Dat mRNA or Dopamine Neuron Subtype Markers NeuroD6 and Grp

**FIGURE 3:**
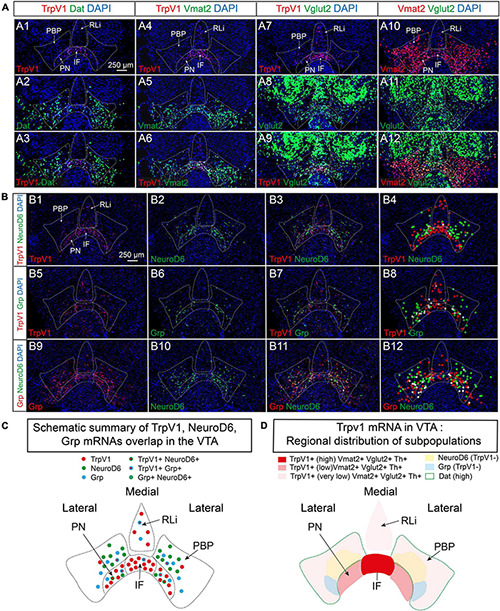
TrpV1 mRNA forms a unique distribution pattern in the medial VTA and co-localizes strongly with Vmat2 but not with Dat, NeuroD6, or Grp mRNAs. **(A,B)** Fluorescent *in situ* hybridization (FISH) was analyzed in coronal VTA sections at postnatal day 3 (P3). DAPI is used for the detection of cell nuclei. **(A)** Left panel: TrpV1 **(A1)**, Dat **(A2)**, TrpV1/Dat **(A3)**. A left-middle panel: TrpV1 **(A4)**, Vmat2 **(A5)**, and TrpV1/Vmat2 **(A6)**. A right-middle panel: TrpV1 **(A7)**, Vglut2 **(A8)**, and TrpV1/Vglut2 **(A9)**. A right panel: Vmat2 **(A10)**, Vglut2 **(A11)**, and Vmat2/Vglut2 **(A12)**. **(B)** A top panel: TrpV1 **(B1)**, NeuroD6 **(B2)**, TrpV1/NeuroD6 **(B3)**, TrpV1/NeuroD6 (**B4**, positive cells encircled). A middle panel: TrpV1 **(B5)**, Grp **(B6)**, TrpV1/Grp **(B7)**, TrpV1/Grp (**B8**, positive cells encircled). A bottom panel: Grp **(B9)**, NeuroD6 **(B10)**, Grp/NeuroD6 **(B11)**, and Grp/NeuroD6 (**B12**, positive cells encircled). Red circles label the red fluorophore, green circles label the green fluorophore, white circles label co-labeling both fluorophores. **(C)** A schematic summary of results obtained in **(B)** outlining TrpV1-positive cells and co-labeling (or its absence) TrpV1, Grp, and NeuroD6 mRNAs in the VTA. **(D)** A schematic summary of results obtained in **(A,B)**, and Figure 1, outlining the main distribution of areas positive for TrpV1 mRNA and co-labeling (or its absence) with Th, Vmat2, Vglut2, Dat, NeuroD6, and Grp mRNAs in the VTA. Scale bars, 250 μm. See Table 1 for cell counting. IF, interfascicular nucleus; PN, paranigral nucleus; PBP, parabrachial pigmented nucleus; RLi, rostral linear nucleus; VTA, ventral tegmental area.

As the substantial co-localization of TrpV1 with Th and Vglut2 points to a strong DA-GLU identity at P3, this was further assessed by analysis of additional components essential to the DA machinery. Dat and Vmat2 mRNAs, encoding the proteins required for DA reuptake (DAT) and vesicular DA transport (VMAT2), were assessed for co-localization with TrpV1 mRNA using FISH in serial sections at P3. In accordance with the reported lateral^*high*^–medial^*low*^ distribution pattern of VTA neurons positive for Dat mRNA (Lammel et al., 2008; Papathanou et al., 2018) and the herein described opposite pattern for TrpV1 mRNA (medial^*high*^ – lateral^*low*^); very few cells showed TrpV1/Dat co-labeling (Figures 3A1–A3 and Table 1). In contrast, in accordance with the broader distribution of Vmat2 than Dat mRNA in the VTA, a clear TrpV1/Vmat2 overlap was identified in all VTA subnuclei positive for TrpV1, except in the RLi (Figures 3A4–A6, Supplementary Figure 3, and Table 1). In IF and PN subnuclei, over 70% of TrpV1^+^ cells were Vmat2^+^. In PBP, containing substantially fewer TrpV1^+^ neurons, over 90% colocalization TrpV1/Vmat2 was observed, in accordance with the strong DA phenotype in this VTA subnucleus (Table 1).

Next, based on the strong TrpV1/Th/Vglut2 and TrpV1/Vmat2 overlap, the extent of Vmat2/Vglut2 overlap was assessed. Confirming data above, TrpV1 and Vglut2 mRNAs highly co-localized in the VTA, primarily in the medial aspect (Figures 3A7–A9). A similar pattern of medial co-labeling was observed between Vglut2 and Vmat2 mRNAs, albeit in a larger proportion of cells than TrpV1 and Vglut2 (Figures 3A10–A12). This is in accordance with the observation of a higher abundance of Vmat2 and Vglut2 mRNAs than TrpV1 mRNA in the medial VTA. Furthermore, Vmat2 mRNA was far less abundant than Vglut2 in RLi, in accordance with the low levels of Th mRNA in this medial subnucleus (Figures 3A10–A12). Instead, most Vglut2/Vmat2 co-localization was detected in the IF and medial PN (Figures 3A10–A12); the subnuclei that are most positive for TrpV1 mRNA.

Having identified that the majority of TrpV1^+^ VTA neurons are positive for the two mRNAs that encode the vesicular transporters essential for the DA-GLU phenotype (VMAT2 and VGLUT2), the hypothesized DA-GLU identity was histologically confirmed. Next, to further define the molecular fingerprint of TrpV1^+^ VTA neurons, additional markers were addressed using CISH and FISH analysis at P3. NeuroD6 and Grp have been identified as molecular markers for medially distributed DA neurons (Chung et al., 2005; Greene et al., 2005; Viereckel et al., 2016; Khan et al., 2017; Kramer et al., 2018; Bimpisidis et al., 2019). Here, it was of interest to find out if TrpV1^+^ VTA neurons form a subgroup within any of these recently described subtypes, or if TrpV1^+^ VTA neurons form a distinct subpopulation.

First, using CISH analysis in serial sections throughout the mesencephalic-hypothalamic area, the pattern of TrpV1 was compared to that of NeuroD6 and Grp mRNAs, using Th as reference for the VTA area (Supplementary Figure 4). NeuroD6 mRNA was most prominent in the PN and PBP subareas and was also found in the RM, but not more than in sparse cells, in the PHA, or the other VTA subareas (IF, RLi, and CLi). Grp mRNA was detected in the IF and PN primarily and was also detected in the lateral PBP and CLi. Grp was also detected in sparse cells in PHA and RM. Some Grp^+^ cells were detected in the SNc (Supplementary Figure 4).

Given this distribution pattern within the VTA, it was of interest to discern if there was any co-localization between TrpV1, NeuroD6, and Grp mRNAs. With the above-shown low NeuroD6 mRNAs levels in VTA subnuclei most positive for TrpV1 mRNA, FISH analysis confirmed a low level of TrpV1/NeuroD6 co-labeling (Figures 3B1–B4 and Table 1). PBP contained some TrpV1/NeuroD6 double-positive cells, corresponding to 20% of TrpV1 cells in this area. The IF and PN, both largely devoid of NeuroD6, showed less than 10% TrpV1/NeuroD6 co-labeling. Furthermore, despite the seemingly similar distribution of TrpV1 and Grp in IF and PN using CISH, analysis using FISH to enable co-localization analysis showed rather modest TrpV1/Grp co-labeling (Figures 3B5–B8 and Table 1). No, or very little, co-labeling of TrpV1 with either NeuroD6 or Grp was observed in either the RLi and CLi, areas where TrpV1 did also not co-localize with Dat (Table 1). Furthermore, NeuroD6 and Grp were not abundantly co-detected but co-localized to some degree in the PN and PBP (Figures 3B9–B12). This co-labeling analysis found that TrpV1, NeuroD6, and Grp mRNAs, that, in gross single-channel CISH observation, showed a similar scattered distribution within the VTA, actually represent largely different VTA neurons, with each mRNA displayed in a unique distribution pattern (Figures 3B1–B12, illustrated in 3C).

To summarize these observations of the P3 mouse brain, TrpV1 mRNA is primarily detected in the IF of the medial VTA, followed by RLi, CLi, PN, and PBP. The molecular identity of TrpV1^+^ VTA neurons includes both DA markers Th and Vmat2 and GLU marker Vglut2, thus defining a TrpV1^+^/Th^+^/Vglut2^+^/Vmat2^+^ subpopulation of DA-GLU neurons. Furthermore, sparse PBP neurons show a TrpV1^+^/Th^+^/Vglut2^–^/Vmat2^+^ (DA) phenotype, while RLi contains ample TrpV1^+^/Th^–^/Vglut2^+^/Vmat2^–^ (GLU) neurons. TrpV1^+^ neurons are generally low in Dat mRNA and are largely distinct from those positive for NeuroD6 and Grp (Figure 3D).

### TrpV1^+^ Neurons of the VTA and Hypothalamus Project Primarily to Limbic Brain Areas

**FIGURE 4:**
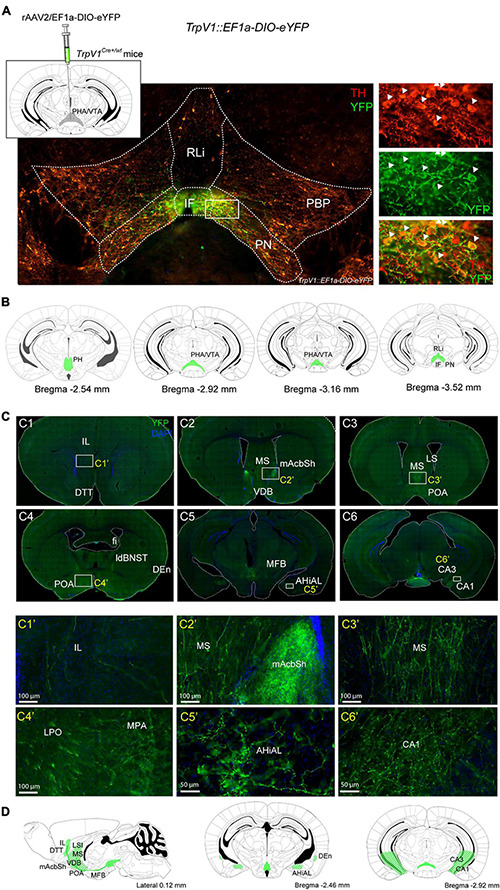
Projections from TrpV1^+^ cells reach multiple forebrain target areas, including nucleus accumbens. **(A)** Top, left: Illustration of virus injection. *TrpV1^*Cre*+/wt^* mice were unilaterally injected with the AAV-EF1a-DIO-eYFP virus to reach the TrpV1-positive area in the posterior hypothalamic nucleus (PHA) and ventral tegmental area (VTA). Bottom: Verification of injection in *TrpV1::EF1a-DIO-eYFP* mice (age postinjection, 13–18 weeks). YFP (green) and TH (red) immunofluorescence in coronal vibratome slice, double-positive cells detected primarily in IF and medial PN of the VTA, close-up in the right-side panel. **(B)** Illustration of coronal sections visualizing sites identified as positive for YFP-positive cell bodies (green fields) along the rostro-caudal axis. **(C)** YFP immunofluorescence in projections reaching various target areas, representative examples are shown. **(C1’–C6’)** Close up of areas shown in white squares in **(C1–C6)**. **(D)** Illustration-visualizing sites identified as positive for YFP-positive projections (green/white stripes in fields). Scale bars 100; 50 μm. AHiAL, a lateral part of the amydalohippocampal area; CA1, field C1 of the hippocampus; CA3, field C3 of the hippocampus; DEn, dorsal endopirifom claustrum; DTT, dorsal tenia tegmental; fi, fimbria of the hippocampus; IF, interfascicular nucleus; ldBNST, a lateral dorsal part of the bed nucleus of the stria terminalis; LPO, lateral preoptic area; LS, lateral septum; MFB, medial forebrain bundle; mNAcSh, nucleus accumbens medial shell; MPA, medial preoptic area; MS, medial septum; PHA, posterior hypothalamic nucleus; PH, posterior hypothalamus; PN, paranigral nucleus of the ventral tegmental area; POA, preoptic areas; RLi, rostral linear nucleus; VDB, the nucleus of the vertical limb of the diagonal band.

A *TrpV1*^*tm1(cre)Bbm*^ (abbreviated *TrpV1*^*Cre/wt*^) transgenic mouse line drives expression of floxed alleles in the hypothalamic-mesencephalic area as demonstrated by analysis of several Cre-driven floxed reporters (Cavanaugh et al., 2011). Here, we took advantage of this validated transgene to address the projection pattern of the identified TrpV1^+^ neuronal population. Placed into a stereotactic frame, adult *TrpV1^*Cre/wt*^* mice were unilaterally injected into the PHA/rostral VTA with an adeno-associated virus (AAV) to enable Cre-driven expression of a floxed construct, encoding the enhanced yellow fluorescent protein (eYFP) (rAAV2/EF1a-DIO-eYFP) (Figure 4A). To validate the injection strategy, brain sections throughout the PHA-VTA area that originated from such injected mice (here referred to as *TrpV1: EF1a-DIO-eYFP* mice) were analyzed for cellular YFP immunofluorescence. YFP^+^ cell bodies were distinct, but sparse, throughout the PHA-VTA area (Figures 4A,B). In accordance with the histological mapping above, the densest YFP^+^ cellular population was observed in the medial location, encompassing the PHA and RM of the caudal hypothalamus, and the IF and medial PN of the VTA (Figures 4A,B). Sparsely distributed YFP^+^ cells were observed in the RLi, PBP, and lateral aspects of the PN (Figures 4A,B). YFP^+^ cells were detected as a string-of-pearl-like band across the PHA-VTA area, similar as described in the original publication showing reporter expression driven by the same *TrpV1^*Cre/wt*^* transgene (Cavanaugh et al., 2011).

Next, YFP^+^ projections and target areas were addressed throughout the brain. Multiple positive sites were identified, primarily areas associated with limbic functions. YFP^+^ projections were detected within the median forebrain bundle. Several known target structures of the PHA/RM and VTA were identified as positive for YFP^+^ fibers. These included mAcbSh; septal, amygdalohippocampal, and preoptic areas; fimbria, CA3, and CA1 fields of the hippocampus; endopiriform nucleus (dorsal); bed nucleus of stria terminalis (dorsolateral) (Figures 4C1–C6’,D). Within the septal area, the medial and lateral septa, the nucleus of the vertical limb of the diagonal band, and the septal hypothalamic nucleus were identified as positive for YFP fibers. Within the preoptic areas, the medial, median, and lateral preoptic areas were identified as positive (Figures 4C1–C6’,D). While target areas reflect projections originating from TrpV1^+^ cells in both VTA and PHA/RM, projections to mAcbSh are likely to originate from the medial VTA, as are sparse YFP-positive projections in the infralimbic and orbital cortices (Figures 4C1–C6’,D).

In summary, by using a *TrpV1: EF1a-DIO-eYFP* strategy with viral injection into the PHA/VTA, scattered YFP^+^ cell bodies were confirmed in the IF, PN, PBP, and RLi of the VTA as well as in the PHA and RM of the caudal hypothalamus. Furthermore, projections from YFP^+^ cells were identified reaching several limbic forebrain areas.

### *TrpV1*^*Cre*^-Driven Targeted Deletion of Vmat2 Causes Its Selective Abrogation in TrpV1^+^ DA and TrpV1^+^ DA-GLU Neurons

To assess if TrpV1^+^ DA-GLU and DA neurons (defined by TrpV1^+^/Th^+^/Vglut2^+^/Vmat2^+^ and TrpV1^+^/Th^+^/Vglut2^–^/Vmat2^+^) contribute to behaviors associated with VTA DA neurons, *Vmat2* gene expression was selectively abrogated in TrpV1^+^ neurons by the generation of a new cKO mouse line (referred to as the *TrpV1^*Cre*^; Vmat2^*flox/flox*^* cKO mouse line). Since the VMAT2 protein is essential for packaging monoamines (including DA) into presynaptic vesicles, *Vmat2* gene-targeting will disable neurons from this mechanism, causing a disruption of DA signaling. This has previously been demonstrated using transgenic mice in which Cre recombinase is under control of promoters directing the *Vmat2*-gene-targeting event either to distinct monoamine systems [DA *via* Dat-Cre, 5′HT *via* Sert-Cre, noradrenaline *via* Net-Cre (Narboux-Nême et al., 2011; Isingrini et al., 2016, 2017)] or to distinct VTA subpopulations [NeuroD6 *via* NeuroD6(NEX)-Cre, Calbindin2/Calretinin/Calb2 *via* Calb2-Cre (Bimpisidis et al., 2019; König et al., 2020)]. As the majority of VTA TrpV1^+^ neurons are Vmat2^+^ and show a dopaminergic phenotype (either as DA-GLU, defined by TrpV1^+^/Th^+^/Vglut2^+^/Vmat2^+^, or DA but not GLU, defined by TrpV1^+^/Th^+^/Vglut2^–^/Vmat2^+^), the knockout of the *Vmat2* gene selectively in TrpV1^+^ neurons will allow assessment of behaviors disturbed by this perturbation of dopaminergic function. Notably, TrpV1-negative Vmat2^+^ monoamine neurons throughout the brain should remain unaffected, as should Vmat2-negative TrpV1^+^ GLU neurons (TrpV1^+^/Th^–^/Vglut2^+^/Vmat2^–^).

**FIGURE 5:**
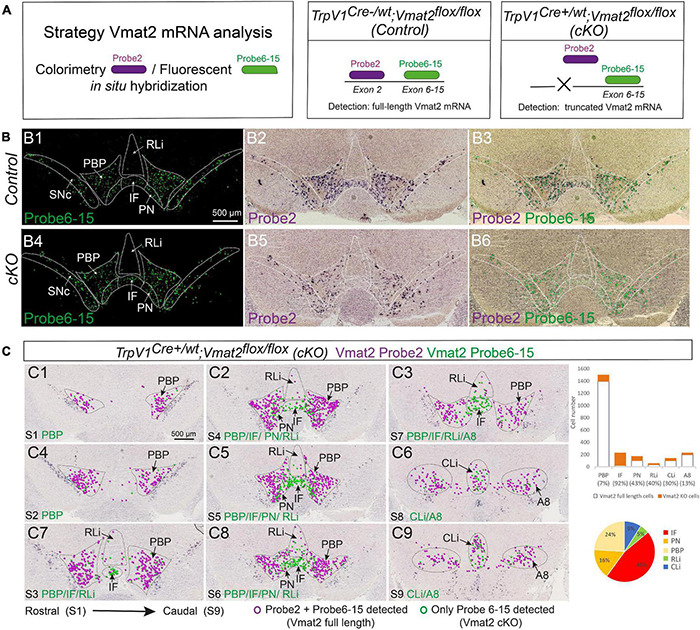
*TrpV1^*Cre*+/wt^;Vmat2^*flox/flox*^* cKO mice show substantial deletion of full-length Vmat2 mRNA in the medial VTA. Analysis of age-matched *TrpV1^*Cre–/wt*^;Vmat2^*flox/flox*^*control and *TrpV1^*Cre*+/wt^;Vmat2^*flox/flox*^*cKO adult mice (12 weeks old). **(A)** Schematic illustration of the Vmat2-two-probe approach used to distinguish the full-length wild-type Vmat2 mRNA (binding Probe 2 and Probe 6–15) from the abrogated Vmat2 mRNA (binding only Probe 6–15) generated in *TrpV1^*Cre*+/wt^;Vmat2^*flox/flox*^* cKO mice. **(B)** Vmat2-two-probe approach implemented on VTA brain sections from *TrpV1^*Cre–/wt*^;Vmat2^*flox/flox*^* control (Control, a top panel) and *TrpV1^*Cre*+/wt^;Vmat2^*flox/flox*^* (cKO, a bottom panel) mice: Vmat2 Probe 2 **(B1,B4)**; Vmat2 Probe 2 combined with Probe 6–15 **(B2,B5)**; same image with colored circles indicating labeled cells **(B3,B6)**. **(C)** Serial sections throughout the VTA area [**C1–C9**, rostral (Section Level 1, S1)] to caudal (Section Level 9, S9) of representative cKO mouse brain for a full assessment of cells positive for wild-type Vmat2 (Probe 2 and Probe 6–15) mRNA and cKOVmat2 mRNA (Probe 6–15 only) as detected by the Vmat2 two-probe approach; the result of cell counting shown in a bar graph (% cells positive for Vmat2 Probe 6–15 in each VTA subnucleus indicated in legend) and a pie chart (distribution of Vmat2cKO cells throughout VTA subnuclei). *N* = 3 mice per genotype and detection, serial sections throughout the brain. Green circles, Vmat2 cKO cells; purple circles, Vmat2 wild-type cells. Scale bars, 500 μm. See Supplementary Figure 5 and Supplementary Table 1 for more details. A8, A8 dopamine area; cKO, conditional knockout; IF, interfascicular nucleus; PBP, parabrachial pigmented nucleus; PN, paranigral nucleus; RLi, rostral linear nucleus; SNc, substantia nigra *pars compacta*; VTA, ventral tegmental area.

By breeding *TrpV1^*Cre*/*wt*^* mice with *Vmat2*^*flox/flox*^ mice in which *exon 2* of the *Vmat2* gene is surrounded by *LoxP* sites (Narboux-Nême et al., 2011), *TrpV1^*Cre*+/wt^; Vmat2^*flox/flox*^* (cKO) mice and *TrpV1^*Cre–/wt*^; Vmat2^*flox/flox*^* (control) mice were generated as littermates in the *TrpV1^*Cre*^; Vmat2^*flox/flox*^* cKO mouse line. All mice were genotyped by PCR. To confirm the *Vmat2*-targeting event, a two-probe strategy was implemented to allow the distinction of Vmat2 full-length mRNA from that of truncated Vmat2 mRNA due to the conditional gene targeting (Figure 5A). Using this strategy, wild-type Vmat2 mRNA (full length) should be detected by binding of two probes (Probe 2 binding to mRNA derived from *Vmat2 gene exon 2*, Probe 6-15 binding to mRNA derived from *Vmat2 gene exons 6-15*). In contrast, Vmat2 cKO mRNA, containing a truncated mRNA due to the targeted deletion of *Vmat2 exon 2*, should fail to bind Probe 2 and, instead, only bind Probe 6-15. In CISH/FISH analysis, binding of both probes will result in a purple/green color precipitate (Vmat2 wild-type mRNA) while binding of only Probe 6-15 will display as fluorescent green color (Vmat2 cKO mRNA) (Figure 5A). Green-only cells thus define the Vmat2 cKO phenotype, and purple/green cells define Vmat2 undisturbed by the targeting event, i.e., wild-type Vmat2 mRNA.

Upon implementing the two-probe strategy in brain sections from adult mice genotyped as control and cKO mice, control mice showed the expected normal distribution of wild-type Vmat2 mRNA labeling (co-localization of both Vmat2 probes) in all monoaminergic brain areas, including VTA and SNc (Figures 5B1–B3 and Supplementary Figure 5).

When addressing cKO mice, it was clear that all monoaminergic systems (including raphe nuclei and noradrenergic, as well as adrenergic cells) outside the VTA and adjacent A8 area were positive for both Vmat2 probes, showing a Vmat2 wild-type phenotype (Supplementary Table 1). However, distributed within VTA subnuclei was a clear density of cells positive only for Vmat2 Probe 6-15, thereby identifying Vmat2 cKO cells (Figures 5B4–B6).

Analysis of serial sections throughout the midbrain showed that the distribution of Vmat2 cKO cells was similar to the described distribution of TrpV1 mRNA with a higher density medially. In the IF, 92% of all Vmat2 mRNA was represented by binding the Vmat2 Probe 6-15 only, thus representing cKO cells, while the remaining 8% were detected by both Vmat2 probes. The Vmat2 cKO phenotype is thereby nearly complete in the IF subnucleus of the VTA. Furthermore, 46% of all Vmat2 cKO cells were found in the IF (Figure 5C and Supplementary Table 1). Thus, in accordance with the highest abundance of TrpV1^+^ neurons of the dopaminergic phenotype (Th, Vmat2) in the IF subnucleus, most Vmat2 cKO cells were found here. Other subnuclei showed a variable density of Vmat2 cKO cells, in accordance with the level of TrpV1, Vmat2, and their co-localization. For example, PBP, which is strongly positive for Vmat2 but sparse for TrpV1, contained 7% Vmat2 cKO cells, while PN, which is positive for both Vmat2 and TrpV1 (but to a lesser degree than IF), contained 43% cKO cells. About 16% of all Vmat2 cKO cells were found in the PN. The linear nuclei (RLi and CLi) represented a smaller proportion of cKO cells, in accordance with the lower abundance of co-localization of Vmat2 and TrpV1 (Figure 5C and Supplementary Table 1).

Following through with triple-probe FISH analysis using the Vmat2 Probe 2 and Vmat2 Probe 6-15 in combination with the Th probe, most VTA Vmat2 neurons (both wild-type and cKO neurons in the cKO mice) were positive for Th mRNA, supporting a dopaminergic phenotype of *Vmat2*-gene-targeted cells (Supplementary Figure 5). Furthermore, to find out more about the timing of the onset of TrpV1^*Cre*^-driven *Vmat2* gene targeting, E15.5 control, and cKO embryos were addressed. Sparse but distinct Vmat2 cKO cells were detected at E15.5 (Supplementary Figure 5), demonstrating the onset of *Vmat2* gene targeting during embryogenesis.

**FIGURE 6:**
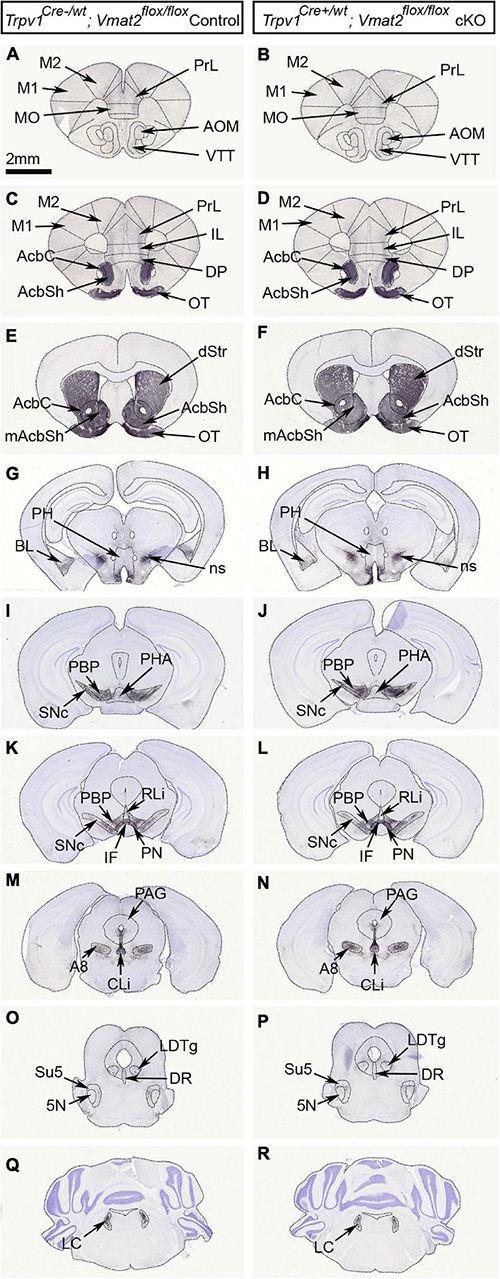
Gross anatomical evaluation reveals intact monoaminergic neurons in *TrpV1^*Cre*+/wt^;Vmat2^*flox/flox*^cKO mice.* Analysis of age-matched *TrpV1^*Cre–/wt*^;Vmat2^*flox/flox*^*control and *TrpV1^*Cre*+/wt^;Vmat2^*flox/flox*^*cKO adult mice (24 weeks). Coronal serial brain sections showing the distribution of tyrosine hydroxylase (TH) immunoreactivity in the midbrain dopamine system; midbrain (VTA subnuclei and SNc) and its target areas in *TrpV1^*Cre–/wt*^;Vmat2^*flox/flox*^*control **(A,C,E,G,I,K,M,O,Q)** and *TrpV1^*Cre*+/wt^;Vmat2^*flox/flox*^*cKO **(B,D,F,H,J,L,N,P,R)** mice. Also shown are additional monoamine populations, including the LC and DR. 5N, motor trigeminal nucleus; A8, A8 dopamine cells of the retrorubral field; AcbC, nucleus accumbens core; AcbSh, nucleus accumbens shell; AOM, anterior olfactory area medial part; BL, basolateral amygdaloid nucleus; CLi, caudal linear nucleus; DP, dorsal peduncular cortex; DR, dorsal raphe nucleus; dStr, dorsal striatum; IF, interfascicular nucleus; IL, infralimbic cortex; LC, locus coeruleus; LDTg, laterodorsal tegmental nucleus; M1, primary motor cortex; M2, secondary motor cortex; mAcbSh, medial accumbens shell; MO, medial orbital cortex; ns, nigrostriatal tract; OT, olfactory tubercle; PAG, periaqueductal gray; PBP, parabrachial pigmented nucleus; PH, posterior hypothalamus; PHA, posterior hypothalamus nucleus; PN, paranigral nucleus; PrL, prelimbic cortex; RLi, rostral linear nucleus; SNc, substantia nigra *pars compacta*; Su5, supratrigeminal nucleus; VTA, ventral tegmental area; VTT, ventral tenia tecta.

Finally, TH immunoreactivity was assessed in adult mice to validate the histological integrity of the midbrain DA system in the absence of normal levels of Vmat2 mRNA from development onwards in TrpV1^+^ VTA neurons. No difference between control and cKO mice could be detected either within the VTA or any of the projection target areas of VTA neurons. Furthermore, no difference between genotypes was observed in the SNc or any other monoaminergic (VMAT2^+^) system as detected by TH immunoreactivity, including the locus coeruleus and dorsal raphe. Thus, the gross anatomy of the DA system as detected histologically by TH immunohistochemistry remained intact despite the abrogation of *Vmat2* gene expression in TrpV1^+^ VTA neurons (Figure 6).

In summary, by generating a new cKO mouse line in which the *Vmat2* gene is conditionally targeted in *TrpV1*^*Cre*^-positive neurons, all VTA subnuclei contain a certain proportion of Th^+^ neurons that lack normal *Vmat2* gene expression levels. By far, the highest proportion of these cKO cells is medially located and primarily distributed within the IF. Next, behavioral assessments were carried out to determine if this genetic manipulation caused any measurable deficits in behavioral capacity.

### *TrpV1^*Cre*+/wt^; Vmat2^*flox/flox*^* Conditional Knockout Mice Show No or Modest Behavioral Alteration in the Open Field and Elevated Plus Maze Paradigms

**FIGURE 7:**
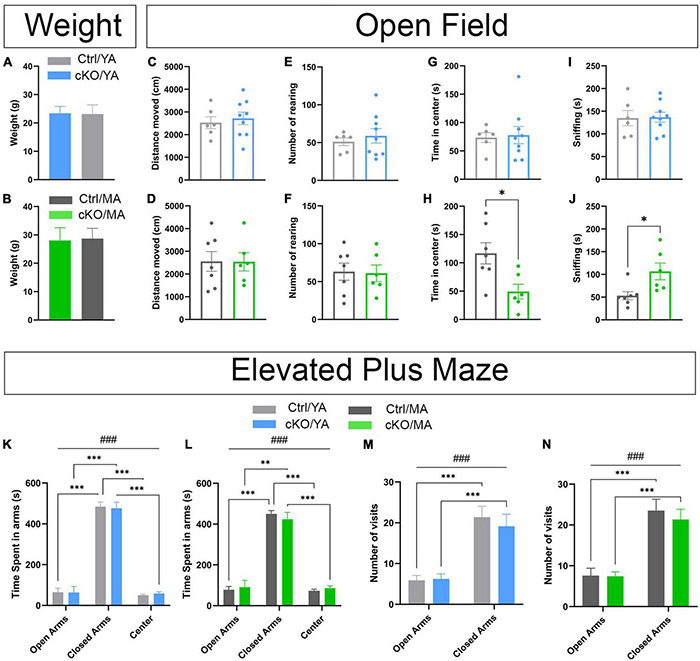
No or modest genotype-dependent alterations in basal behavior were displayed by *TrpV1^*Cre*+/wt^;Vmat2^*flox/flox*^*cKO mice. Analysis and comparison of age-matched *TrpV1^*Cre–/wt*^;Vmat2^*flox/flox*^control (Ctrl)* and *TrpV1^*Cre*+/wt^;Vmat2^*flox/flox*^* conditional knockout (cKO) mice at young adult (YA; 8 weeks old) and mature adult (MA; 18 weeks old) age. **(A,B)** Weight at the beginning of an amphetamine sensitization paradigm (**A**, YA; **B**, MA). **(C–J)** An open field test. Ctrl/YA (*N* = 6) and cKO/YA (*N* = 9), Ctrl/MA (*N* = 7), and cKO/MA (*N* = 6) data are expressed as mean ± SEM. **(C)** distance-moved YA. **(D)** distance-moved MA. **(E)** Number of rearing YA. **(F)** Number of rearing MA. **(G)** Time spent in center YA. **(H)** Time spent in center MA (*p = .0154, Ctrl/MA vs. cKO/MA). **(I)** sniffing YA. **(J)** sniffing MA (*p = 0.0193, Ctrl/MA vs. cKO/MA). **(K–N)** Elevated plus maze (EPM). Ctrl/YA (*N* = 8) and cKO/YA (*N* = 13), Ctrl/MA (*N* = 11) and cKO/MA (*N* = 12), data are expressed as mean ± SEM. **(K)** time spent in arms YA (###*p* < 0.001, arms effect; ***p < 0.001, Closed Arms vs. Open Arms and Center). **(L)** Time spent in arms MA (###*p* < 0.001, arms effect; ***p < 0.001, Closed Arms vs. Open Arms and Center; **p = 0.001, Closed Arms vs. Open Arms). **(M)** Number of visits in arms YA (###*p* < 0.001, arms effect; ***p < 0.001, Closed Arms vs. Open Arms). **(N)** Number of visits in arms MA (###*p* < 0.001, arms effect; ***p < 0.001, Closed Arms vs. Open Arms).

By observation of the mice in their home-cage environment, there was no apparent difference between *TrpV1^*Cre–/wt*^; Vmat2*^*flox/flox*^ control and *TrpV1^*Cre*+/wt^;Vmat2^*flox/flox*^* cKO mice in the way they moved around, interacted, or fed. Thus, caretaker inspection revealed no difference between control and cKO mice. Throughout subsequent analyses, age-matched control and cKO mice of two age groups [Young adult (YA) mice, 8 weeks of age, and mature adult (MA) mice, 18 weeks of age, at the start of behavioral analyses] were assessed to determine if any age-related progression in the behavioral display could be detected (Supplementary File 1; statistical details of all behavior analyses). First, regular weight measures confirmed that control and cKO mice increased their body weight at a similar rate throughout their first weeks (Supplementary Figure 6). At 8 and 18 weeks of age (when behavior experiments started), there was no difference in weights between genotypes of either age group (Figures 7A,B).

To ascertain basal locomotor and exploratory activities, mice were analyzed in the open field test. Multiple parameters relevant to vertical and horizontal movement, exploratory visits to different areas in the open field chamber, and bodily arrangements, such as self-grooming, contraction, and sniffing, were analyzed (Figures 7C,E,G,I and Supplementary Figure 7). No major difference in either of these behaviors was detected in either YA (Figures 7C–F and Supplementary Figure 7) or MA (Figures 7D,F,H,J and Supplementary Figure 7) cKO mice compared with control mice. However, while locomotor parameters (distance moved and rearing) were similar between cKO and control mice of both age groups (Figures 7C–F), there was a difference in the time spent in the center of the arena in the MA, but not YA, age group (Figures 7G,H). MA cKO mice spent significantly less time in the center than their corresponding control group, showing a profile that more looked like YA control and cKO mice than MA control mice. Also sniffing was different between the genotype groups, but only in the MA age group (Figures 7I,J). Overall, MA control mice showed increased time in the center and decreased their sniffing compared with MA cKO mice, but this difference was not shown in the YA group (Figures 7G,J).

To ascertain if these observations were correlated with anxiety, mice were analyzed in the elevated plus maze (EPM). This maze consists of four arms elevated from the floor, two arms sheltered (closed), and two arms open. Generally, mice explore the whole maze but prefer the sheltered areas and avoid the open arms. An anxious phenotype is defined by a heightened stay in the closed arms due to avoidance of the open arms, while an anxiolytic phenotype shows an increased preference for the open arms and increased number of visits around the arena. Comparing cKO and control mice, no difference in their behavior in the EPM was observed (Figures 7K–N and Supplementary Figure 7). All mice spent significantly more time in the closed than open arms (Figures 7K,L). There was no difference between cKO and control mice at any age. Mice also moved around the maze at a similar amount, with no significant differences in visits to any arena between genotype groups at any age (Figures 7M,N). Thus, no genotype-dependent display of anxiety (avoidance or preference phenotype) was confirmed in the EPM. Instead, among ample parameters analyzed, increased sniffing and reduced explorations manifested by mature cKO mice in the open field were the only differences detected between cKO and control mice.

In summary, quantified behavioral data in the open field and EPM paradigms along with caretakers observations demonstrate that *TrpV1^*Cre*+/wt^;Vmat2^*flox/flox*^* cKO mice are largely indistinguishable from control mice, regardless of age.

### An Amphetamine Sensitization Paradigm Reveals a “Pre-sensitized” Phenotype of *TrpV1^*Cre*+/wt^;Vmat2^*flox/flox*^* Conditional Knockout Mice

VTA DA neurons have long been associated with many different aspects of reward processing correlated with DA release in limbic and cognitive forebrain target areas (Berridge and Robinson, 1998; Di Chiara, 1998; Salamone and Correa, 2012; Schultz, 2016). For example, DA release is a critical aspect of psychostimulant response and can be detected as increased locomotion, often referred to as psychomotor behavior. The concept of behavioral sensitization refers to a progressively greater and enduring behavioral response (including locomotion) that occurs following repeated stimulant administration. This phenomenon has been hypothesized to underlie aspects of human stimulant addiction, as well as several psychiatric conditions (Robinson and Becker, 1986; Robinson, 1993). Lately, also the DA-GLU phenotype of VTA neurons has been associated with psychostimulant-induced responses, with similar implications for addiction and psychiatric conditions (reviewed in Mingote et al., 2017; Morales and Margolis, 2017; Bimpisidis and Wallén-Mackenzie, 2019; Eskenazi et al., 2021). To determine if targeted deletion of VMAT2 selectively in TrpV1^+^ VTA neurons had any consequence for a psychostimulant response, drug-induced locomotor effects were analyzed using an amphetamine sensitization paradigm.

**FIGURE 8:**
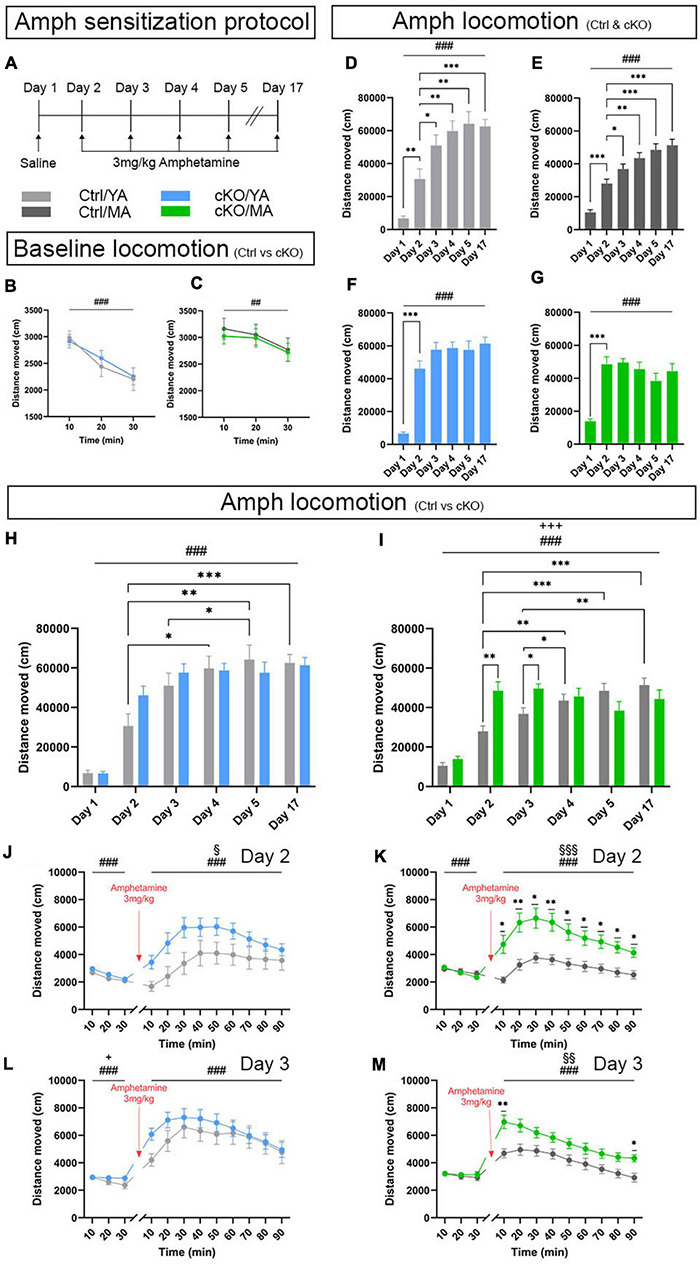
An amphetamine sensitization paradigm identifies altered locomotor response displayed by *TrpV1^*Cre*+/wt^;Vmat2^*flox/flox*^*cKO mice. Analysis and comparison of age-matched *TrpV1^*Cre–/wt*^;Vmat2^*flox/flox*^*control (Ctrl) and *TrpV1^*Cre*+/wt^;Vmat2^*flox/flox*^* conditional knockout (cKO) mice at young adult (YA; 8 weeks old) and mature adult (MA; 18 weeks old) age. **(A)** Amphetamine sensitization protocol. **(B,C)** Locomotor activity during 30 min that preceded saline injection. **(B)** Ctrl/YA (*N* = 14) and cKO/YA (*N* = 22). Data expressed as mean ± SEM (###*p* < 0.001, time effect). **(C)** Ctrl/MA (*N* = 18) and cKO/MA (*N* = 18). Data expressed as mean ± SEM (##*p* = 0.02, time effect). **(D–M)** Amphetamine-induced locomotion, 90 min following amphetamine injection; Ctrl/YA (*N* = 22) and cKO/YA mice (*N* = 22). Ctrl/MA (*N* = 18) and cKO/MA mice (*N* = 18). **(D)** Distance moved presented as mean ± SEM for each session Ctrl/YA (###*p* < 0.001, day effect; ^∗∗^*p* = 0.003, Day 2 vs. Day 1; ^∗^*p* = 0.047, Day 2 vs. Day 3; ^∗∗^*p* = 0.008, Day 2 vs. Day 4; ^∗∗^*p* = 0.002, Day 2 vs. Day 5; ^∗∗∗^*p* < 0.001, Day 2 vs. Day 17). **(E)** Distance moved presented as mean ± SEM for each session Ctrl/MA (###*p* < 0.001, day effect; ^∗∗∗^*p* < 0.001, Day 2 vs. Day 1; ^∗^*p* = 0.028, Day 2 vs. Day 3; ^∗∗^*p* = 0.002, Day 2 vs. Day 4; ^∗∗∗^*p* < 0.001, Day 2 vs. Day 5; ^∗∗∗^*p* < 0.001, Day 2 vs. Day 17). **(F)** Distance moved presented as mean ± SEM for each session cKO/YA (###*p* < 0.001, day effect; ^∗∗∗^*p* < 0.001, Day 2 vs. Day 1). **(G)** Distance moved presented as mean ± SEM for each session cKO/MA (###*p* < 0.001, day effect; ^∗∗∗^*p* < 0.001, Day 2 vs. Day 1). **(H)** Distance moved presented as mean ± SEM for each session, Ctrl/YA vs. cKO/YA (###*p* < 0.001, day effect; ^∗^*p* = 0.027, Ctrl/YA Day 2 vs. Day 4; ^∗∗^*p* = 0.006, Ctrl/YA Day 2 vs. Day 5; ^∗∗^*p* = 0.016, Ctrl/YA Day 3 vs. Day 5; ^∗∗∗^*p* < 0.001, Ctrl/YA Day 2 vs. Day 17). **(I)** Distance moved presented as mean ± SEM for each session, Ctrl/MA vs. cKO/MA (###*p* < 0.001, day effect; +++*p* < 0.001, day × genotype effect; ^∗∗^*p* = 0.003, Day 2 Ctrl/MA vs. cKO/MA; ^∗^*p* = 0.017, Day 3 Ctrl/MA vs. cKO/MA; ^∗∗^*p* = 0.007, Ctrl/MA Day 2 vs. Day 4; ^∗∗∗^*p* < 0.001, Ctrl/MA Day 2 vs. Day 5; ^∗∗∗^*p* < 0.001, Ctrl/MA Day 2 vs. Day 17; ^∗^*p* = .03, Ctrl/MA Day 3 vs. Day 4; ^∗∗^*p* = 0.001, Ctrl/MA Day 3 vs. Day 4). **(J)** Amphetamine-induced locomotion on Day 2, YA. Distance moved presented as mean ± SEM for each 10-min period (###*p* < 0.001, time effect; §*p* = 0.049, genotype effect). **(K)** Amphetamine-induced locomotion on Day 2, MA. Distance moved presented as mean ± SEM for each 10-min period (###*p* < 0.001, time effect; §§§*p* < 0.001, genotype effect; ^∗^*p* < 0.05, Ctrl/MA vs. cKO/MA; ^∗∗^*p* < 0.01, Ctrl/MA vs. cKO/MA). **(L)** Amphetamine-induced locomotion on Day 3, YA. Distance moved presented as mean ± SEM for each 10-min period (###*p* < 0.001, time effect). **(M)** Amphetamine-induced locomotion on Day 3, MA. Distance moved presented as mean ± SEM for each 10-min period (###*p* < 0.001, time effect; §§*p* = 0.002, genotype effect; ^∗^*p* < 0.05, Ctrl/MA vs. cKO/MA; ^∗∗^*p* < 0.01, Ctrl/MA vs. cKO/MA).

The sensitization protocol lasted 17 days, during which a saline injection was given on Day1, followed by one injection per day of 3-mg/kg amphetamine during 4 days (Days 2, 3, 4, and 5), and a challenging day in which the mice received a last amphetamine injection (Day 17) (Figure 8A). Baseline locomotion was measured on Day 1, prior to the saline injection. No difference between cKO and control mice was observed in either young adult (YA) or mature adult (MA) groups (Figures 8B,C). Furthermore, no sex differences were observed (Supplementary File 1). For all analyses, mice were, therefore, pooled according to genotype (control and cKO) and age (YA and MA).

Upon administration of amphetamine, mice commonly show significantly heightened locomotion (hyperlocomotion) above baseline levels that last for 60–90 min, with a peak around 30–40 min. Such hyperlocomotion was observed. All control mice responded with hyperlocomotion (Figures 8D,E). In both YA and MA control mice, this hyperlocomotion increased progressively and was significantly stronger on each subsequent injection day, an index of sensitization toward the psychostimulant (Figures 8D,E). Curiously, *TrpV1^*Cre*+/wt^;Vmat2^*flox/flox*^* cKO mice showed a different response profile to the drug (Figures 8F,G). Both YA and MA cKO mice did, indeed, show strong hyperlocomotion on Day 2, the first day of amphetamine injection (Figures 8F,G). However, they failed to progressively increase their locomotor response across sessions. This led to a lack of significant difference between Day 2 and Day 5, and also between Day 2 and Day 17, despite repeated exposure to the stimulus. This difference was observed in both the YA and MA cKO groups, demonstrating an absence of the normally observed behavioral sensitization.

When comparing responses between genotype groups (control *vs* cKO), significant differences were evident when amphetamine, but not saline, was administered (Figures 8H,I). Saline injection (Day 1) induced no differences in locomotion between cKO and control mice in any age group. In response to amphetamine, MA cKO mice increased their locomotion above control mice on both Day 2 and Day 3. On Day 2, a tendency for a higher effect on cKO compared with control mice was observed also in the YA group. Thus, cKO mice showed accentuated hyperlocomotion above the level of control mice. For this reason, further analysis was motivated. The locomotor response was assessed by dividing each amphetamine session into 10-min periods (Figures 8J–M and Supplementary Figures 8A–F). The statistical analysis supported the observation of heightened hyperlocomotor response in the cKO groups by showing a genotype effect on Day 2 for both the YA and MA groups (Figures 8J,K). On Day 3, a difference was observed only in MA mice (Figures 8L,M). Upon subsequent injections (Days 4, 5, and 17), both YA and MA cKO mice still achieved hyperlocomotion in response to amphetamine, but the response was similar to that observed in control mice (Figures 8H,I and Supplementary Figures 8A–F).

The accentuated hyperlocomotion displayed by the two cKO groups was thereby observed in the initial (acute) phase of the sensitization paradigm but did not progress further. This initially strong amphetamine-induced behavioral response suggests a “pre-sensitized” phenotype caused by the absence of normal *Vmat2* gene expression levels in selected VTA neurons. cKO mice thus show immediately enhanced hyperlocomotion that control mice reach only after repeated stimuli. The strong initial increase upon a first injection followed by a blunted response curve demonstrates a lack of regular sensitization to amphetamine but suggests an enhanced sensitivity to the drug. Finally, the effect was more pronounced in mature than young cKO mice, suggesting a progression in a phenotype with age.

## Discussion

Major attention has been directed at the role of the TRPV1 channel in the sensory processing of heat, pain, and body temperature, as well as its responsivity to various ligands, such as capsaicin and cannabinoids (Caterina et al., 1997; Szallasi et al., 2007). However, the presence of TRPV1 in the brain has been debated due to the difficulty in pinpointing its detection (Cavanaugh et al., 2011; Ramírez-Barrantes et al., 2016). Here, we present a series of findings that allow us to classify a distinct VTA neuron subtype according to its expression of the *TrpV1* gene. Furthermore, by abrogation of vesicular DA packaging *via* selective targeting of VMAT2 in TrpV1^+^ neurons, we identify a role in mediating the behavioral response to the psychostimulant amphetamine, a substance often clinically prescribed to alleviate symptoms in ADHD but which is also used/misused and can cause addiction.

The VTA is a heterogeneous brain area in which subtypes/subpopulations of neurons today can be distinguished by molecular fingerprints. This advancement is based on recent efforts using microarray and transcriptomics-based methodology which allows for dissociation of neurons beyond neurotransmitter identity (reviewed in Poulin et al., 2020). Here, we demonstrate that expression of the *TrpV1* gene, which we previously detected as elevated in the VTA over SNc in the newborn mouse (Viereckel et al., 2016), represents a distinct marker for certain VTA subpopulations, primarily one that defines DA-GLU neurons positioned close to the midline. Histological mapping across the rostro-caudal axis of the VTA in newborn mice allowed the identification of three classes of neurons that, based on the presence of TrpV1 mRNA, can be defined according to: 1) One main *DA-GLU subpopulation* distinguished by a TrpV1^+^/Th^+^/Vglut2^+^/Vmat2^+^ phenotype, strongly located to the medial VTA (e.g., 49% of TrpV1^+^ VTA cells are in the IF); 2) one small *DA subpopulation* defined by a TrpV1^+^/Th^+^/Vglut2^–^/Vmat2^+^ phenotype present primarily in the PBP; and 3) one small *GLU subpopulation* defined by a TrpV1^+^/Th^–^/Vglut2^+^/Vmat2^–^phenotype present in the RLi at the border to, and continuing into, the PHA and RM. DA-GLU neurons located in the IF are largely positive for TrpV1 mRNA (85%), while other more rarely occurring DA-GLU neurons, for example, in the laterally positioned SNc, are TrpV1-negative. Thus, TrpV1 mRNA distinguishes a medial subpopulation of DA-GLU neurons.

Further defining the molecular properties of the TrpV1^+^ phenotype, we can conclude that it is largely distinct from VTA DA subpopulations defined by NeuroD6 or Grp, described in several recent reports (Viereckel et al., 2016; Khan et al., 2017; Kramer et al., 2018; Bimpisidis et al., 2019). Both NeuroD6 and Grp mRNAs show a similar level of scattered distribution within the VTA as TrpV1. However, by implementing fluorescent co-localization analysis throughout the VTA, a non-overlapping distribution could be revealed. The medial position of TrpV1^+^ VTA neurons is further emphasized by their low level of Dat mRNA, a property primarily of lateral midbrain DA neurons, as well as by the altered locomotor response to amphetamine of mice gene targeted for *Vmat2* in TrpV1^+^ VTA neurons (further discussed below).

Parallel to the specific spatial distribution and molecular identity of TrpV1^+^ neurons, a striking temporal regulation of TrpV1 is revealed in the mouse brain. TrpV1 mRNA is detected at E14.5 and P3, while almost no TrpV1 mRNA can be detected 9 days later (P12), yet alone in adulthood. A previous study showed its absence at E11.5 and earlier, the time point when DA neurons start differentiating and express both *Th* and *Vglut2* genes (Dumas and Wallén-Mackenzie, 2019). Together, these results demonstrate a temporal curve, with a TrpV1 peak in the developing VTA around E14.5-P3. Lower TrpV1 mRNA levels in adolescent and adult mice than in perinatal mice likely are correlated with the reported difficulties with detection in adult rodents, as discussed in several studies (see references in Cavanaugh et al., 2011; Ramírez-Barrantes et al., 2016). Furthermore, low detection levels in adult mice might contribute to an underestimate of the presence of TrpV1 mRNA in the mouse brain. Temporal regulation of gene expression is a common feature, not unique to *TrpV1*. It follows that histological features defined by addressing multiple gene expression patterns in the brain of newborn mice might not show the same co-localization patterns at any other age but represent snap shots of the given time point. However, the temporal regulation is of particular interest from a functional point of view, tentatively supporting the idea of a role for TRPV1 in the perinatal function of the midbrain-forebrain area that should be of interest to future study. While not addressing any such role for the TRPV1 protein here, pharmacological studies have already shown that neuronal excitability across the brain, including in DA neurons, is affected upon treatment with TRPV1 ligands, arguing for its presence and function in the brain (Marinelli et al., 2003, 2005, 2007). With the current identification of *TrpV1* gene expression in a restricted set of VTA neurons, functional approaches should be of particular interest to pursue, not least considering the abundance of pharmacological substances available to manipulate the TRPV1 channel.

Despite temporal regulation of the *TrpV1* gene and any reported difficulties with detection of its transcribed and translated products in adulthood, adult *TrpV1*^*Cre/wt*^ transgenic mice can be used to study the mature brain. This was shown already in the original publication (Cavanaugh et al., 2011) and reinforced here. By implementing viral-genetic mapping of projection patterns, a series of limbic structures could be reliably identified as targets of *TrpV1^*Cre*/wt^* VTA/PHA neurons, including septal, hippocampal, and accumbal structures. These likely reflect the sum of projections originating from *TrpV1^*Cre/wt*^*-positive neurons of both the VTA and posterior hypothalamus, thus neurons of DA, GLU, and DA-GLU neurotransmitter phenotype. Using this same strain of *TrpV1^*Cre/wt*^*mice, direct focus on TrpV1^+^ VTA neurons of the dopaminergic phenotype (DA and DA-GLU) was purposefully enabled by taking advantage of a Vmat2 cKO approach. A new mouse line produced, *TrpV1^*Cre*+/wt^;Vmat2^*flox/flox*^* cKO mice showed a distinct lack of full-length Vmat2 mRNA in TrpV1^+^/Vmat2^+^neurons of the VTA, primarily in the IF. However, general behavior was undisturbed by this manipulation. The only differences noted between control and cKO mice in the drug-naïve state were decreased exploration and increased sniffing in mature, but not young, adult cKO mice compared with age-matched control mice. However, these were not confirmed by altered behavior in the elevated plus maze, and a relevant interpretation is, therefore, challenging.

The most striking behavioral finding was, instead, the altered response curve in an amphetamine sensitization paradigm. This experiment was motivated by the association of VTA DA and DA-GLU neurons with amphetamine response. Considering the main neurotransmitter phenotype of TrpV1^+^ VTA neurons identified as DA-GLU (TrpV1^+^/Th^+^/Vglut2^+^/Vmat2^+^), amphetamine was selected to challenge these neurons. The current correlation between DA-GLU neurons and amphetamine-induced locomotion is primarily based on cKO studies of the GLU aspect of the DA-GLU phenotype, either *via* cKO of the *Vglut2* gene (Birgner et al., 2010; Hnasko et al., 2010; Fortin et al., 2012) or the glutamate recycling enzyme glutaminase (gene *Gls1*) (Mingote et al., 2017) in DA neurons. Summarizing several studies targeting *Vglut2* in DAT^+^ neurons (*Vglut2^*lx/lx*^;Slc6a3^*Cre/wt*^*), this kind of manipulation has been shown to cause reduced GLU release and reduced glutamatergic postsynaptic currents accompanied by secondary effects on striatal DA release as well as significant alteration of psychomotor response upon psychostimulant (amphetamine, cocaine) administration (Birgner et al., 2010; Hnasko et al., 2010; Alsiö et al., 2011; Fortin et al., 2012).

Despite these studies, a detailed understanding of how DA-GLU neurons contribute to behavioral regulation is still limited (recently reviewed in Eskenazi et al., 2021). With our identification of a subpopulation of DA-GLU neurons as positive for TrpV1, a new opportunity to further the understanding of this complex neuronal phenotype has been provided. Here, instead of targeting the GLU aspect of the DA-GLU phenotype, we abrogated their DA identity by producing the *TrpV1^*Cre*+/wt^;Vmat2^*flox/flox*^* cKO mice. This was important as both sensory TrpV1^+^ neurons (Lagerström et al., 2010; Scherrer et al., 2010), and those TrpV1^+^ neurons we describe in the hypothalamus, are of glutamatergic identity (Vglut2). Thus, specificity for VTA DA-GLU neurons could never be achieved by using a similar Vglut2 cKO approach (even with *TrpV1^*Cre/wt*^* as a driver) as used in previous studies of DA-GLU neurons. Instead, the current *TrpV1^*Cre*^;Vmat2^*flox/flox*^* cKO approach achieved high specificity for gene targeting of *Vmat2* selectively in TrpV1^+^ neurons of the VTA, as validated by detailed histological analysis throughout the brain. Thus, using the present Vmat2-based approach, in addition to targeting VMAT2 rather than VGLUT2 in DA-GLU neurons, a new level of selectivity for a subgroup of DA-GLU neurons is reached. Building onto the revelation of a TrpV1^+^/Th^+^/Vglut2^+^/Vmat2^+^ phenotype in the medial VTA, and the established association of medial VTA DA and DA-GLU neurons with the psychostimulant response, the current results reveal a role for the TrpV1^+^ subpopulation of DA-GLU neurons in amphetamine response. Heightened locomotion above control levels upon amphetamine injection was observed in both young and mature (YA and MA) *TrpV1^*Cre*+/wt^;Vmat2^*flox/flox*^*cKO mice during the first 1–2 injection days. The effect was stronger in MA cKO mice than in YA cKO mice, suggesting an enhancement of the cKO phenotype with age. Furthermore, all cKO mice, independent of age, show a lack of progressive sensitization upon repeated amphetamine administration.

We reason that the initial robust response to amphetamine might be due to a compensatory postsynaptic effect induced by the inability of TrpV1^+^ neurons to synthesize the VMAT2 protein. With the onset of *Vmat2* gene targeting in TrpV1-Cre-positive cells during embryonic development of the VTA, functional compensations effects likely occur as a consequence of the disrupted VMAT2 function. While not observed as altered levels of TH in the mesolimbic or other monoaminergic systems, a more refined methodology might have identified neurocircuitry alterations. Responses caused by altered VMAT2 levels have been amply reported in the literature. For example, heterozygous VMAT2 KO mice show increased horizontal locomotor activity compared with wild-type littermates in response to acute administration of the drug (Wang et al., 1997). Also, hypomorphic *Vmat2* transgenic mice display “behavioral supersensitivity” to amphetamine (Mooslehner et al., 2001). Knocking out the *Vmat2* gene may induce a redistribution of DA from the vesicles to the cytoplasm, where, under physiological conditions, it is metabolized to DOPAC (3,4-dihydroxyphenylacetic acid) by cytosolic monoamine oxidase, MAO. In addition, VMAT2 has been shown to provide a protective effect from oxidative stress-related damage in neurons (Guillot and Miller, 2009; Lohr et al., 2016). This condition of decreased release of DA in response to action potential could probably induce plastic changes at the postsynaptic level that result in supersensitization toward DA.

Furthermore, based on the knowledge that chronic treatment with reserpine leads to upregulation and sensitization of D1 and D2 receptors (Rubinstein et al., 1990; Neisewander et al., 1991), it has been proposed that the same mechanism underlies amphetamine-induced hyperlocomotion observed in different VMAT2 cKO mice, targeting distinct monoaminergic populations (Isingrini et al., 2016). Thus, similar findings as reported here have been observed in other mouse strains that lack normal VMAT2 levels in the monoamine systems. It is evident that mice lacking normal levels of VMAT2 show an initial heightened response to amphetamine above control levels. This has been shown with both acute injections and with sensitization paradigms. Importantly, it is only the initial responses that are accentuated in such a sensitization paradigm (that is, the acute effect).

By affecting VMAT2 levels (rather than VGLUT2), the impact on response to amphetamine is direct. Amphetamine binds to the VMAT2 protein, reducing the ability of this transporter to refill the vesicles with neurotransmitters and, in turn, increasing cytosolic DA (Sulzer and Rayport, 1990; Sulzer et al., 1995). The level of VMAT2 is crucial both for exocytotic and carrier-mediated release of DA because it regulates the size of the vesicular pool and the concentration of DA in the cytosol (Patel et al., 2003). In addition to VMAT2, amphetamine affects DA levels by inhibiting MAO, thus preventing the metabolism of DA, leading to accumulation of DA in the cytosol (Miller et al., 1980; Brown et al., 2001; Fleckenstein et al., 2007) and by a carrier-reversal release mechanism through the DAT molecule (Giros et al., 1996). Based on this previous knowledge, and the lack of progressive increase in response observed here, it may seem as if the amphetamine effect were blunted in VMAT2 cKO mice. However, in non-physiological conditions (such as upon cKO of VMAT2), when less DA is stored in vesicles, the availability of DA in the cytosol, which can be released through a DAT-mediated modality, may become significant. In addition, the capacity of amphetamine to inhibit MAO (Miller et al., 1980) can momentarily increase the intracellular concentration of DA, which can be released by reverse transport of DAT. Thus, the behavioral augmentation observed in *TrpV1^*Cre*+/wt^;Vmat2^*flox/flox*^*cKO mice upon acute amphetamine administration (initial doses) can be seen as the combined result of a momentarily increased release of DA and the postsynaptic supersensitization due to the absence of VMAT2 protein from prenatal development.

Mice lacking normal VMAT2 levels thus seem to display a “pre-sensitized,” or “super-sensitized” state, which is reflected in their enhanced psychomotor behavior to initial (acute) amphetamine injections. The behavioral phenotype of the cKO mice presented here shows that TrpV1^+^ VTA neurons contribute to this response. Similar to any study implementing knockout methodology induced during brain development, compensatory neurocircuitry mechanisms might contribute to the observed phenotype. All the same, given that alterations in response to amphetamine sensitization are an indicator of dysfunction, the “pre-sensitized phenotype” of mice lacking VMAT2 in the TrpV1 subpopulation of VTA DA and DA-GLU neurons may be of critical importance to addiction and other psychiatric conditions.

To summarize this study, TrpV1 defines a subset of medial VTA neurons which can be associated with behavioral response to the psychostimulant amphetamine. Future studies should be important to fully uncover how the TrpV1 identity contributes to VTA function in normal and pathological conditions.

## Methods Section

### Mice

#### Transgenics, Housing, and Ethical Permits

Mice were housed at the animal facility of Uppsala University (UU) where they had access to food and water *ad libitum* in standard humidity and temperature conditions and lived under a 12-h dark/light cycle. All animal experimental procedures performed at UU (generation and maintenance of mice, viral-genetic tracing, immunohistochemistry, behavior analyses) followed Swedish (Animal Welfare Act SFS 1998:56) and European Union Legislation (Convention ETS 123 and Directive 2010/63/EU) and were approved by the local Uppsala Ethical Committee. Experimental procedures performed at Oramacell, Paris (*in situ* hybridization on tissue derived from UU, maintenance of wild-type mice) were approved by the Regional Ethics Committee No. 3 of Ile-de-France region on Animal Experiments, and followed the guidelines of the European Communities Council Directive (86/809/EEC) and the Ministère de l’Agriculture et de la Forêt, Service Vétérinaire de la Santé et de la Protection Animale (permit No. A 94-028- 21).

A colony of transgenic mice for the study was generated from initial breeding of two *TrpV1^*tm1(cre)Bbm*^* (here abbreviated *TrpV1^*Cre/wt*^*) transgenic male mice purchased from The Jackson Laboratory (stock #017769) and maintained by breeding to female wild type [C57BL/6N (abbreviated Bl6) Taconic]. *TrpV1^*Cre/wt*^*mice were originally produced by, and donated to, The Jackson Laboratory by Dr. Allan Basbaum, University of California, United States. The *TrpV1^*Cre/*+^*mice containing a myc-tagged IRES-cre sequence inserted downstream of the TrpV1 stop codon. The endogenous TrpV1-coding sequence is not disrupted. When bred with a mouse strain containing a *lox*-flanked sequence, Cre-mediated recombination will occur, as previously validated (Cavanaugh et al., 2011). *Vmat2*^*flox/flox*^ mice, in which exon 2 of the *Vmat2* gene is flanked by *LoxP* sites, were originally donated by Dr. Bruno Giros, McGill University, Canada (Narboux-Nême et al., 2011). A new cKO mouse line (*TrpV1^*Cre*^;Vmat2^*flox/flox*^*) was produced for this study by breeding male *TrpV1^*Cre/wt*^* mice with female *Vmat2*^*flox/flox*^ mice, first generating heterozygous mice of which male mice were bred with female *Vmat2*^*flox/flox*^ mice to generate *TrpV1^*Cre*+/wt^;Vmat2^*flox/flox*^* cKO and *TrpV1^*Cre–/wt*^;Vmat2^*flox/flox*^* control mice in the same litter. Littermate mice of both male and female sex were used throughout the analyses.

#### Genotyping

PCR analyses were performed to confirm the genotype of transgenic mice using DNA extracted from ear biopsies.

TrpV1-Cre forward primer: 5′GCGGTCTGGCAGTAAAAA CTATC;TrpV1-Cre reverse primer: 5′GTGAAACAGCATTG CTGTCACTT; Vmat2-Lox forward primer: 5′GACTCAGG GCAGCACAAATCTCC; Vmat2-Lox reverse primer: 5′GAA ACATGAAGGACAACTGGGACCC.

### *In situ* Hybridization Histochemistry

#### Brain Section Preparation

Wild-type Bl6, *TrpV1^*Cre*+/wt^;Vmat2^*flox/flox*^* cKO, and *TrpV1^*Cre–/wt*^;Vmat2^*flox/flox*^* control mice were euthanized, brains dissected, and snap frozen in cold isopentane (2-Methylbutane, (−30°/−35°C). Sections were cut on a cryostat at the 16-μm thickness and kept at −80°C until their use. For a generation of mouse embryos, mice were mated and females checked for a vaginal plug in the morning. The morning of vaginal plus was determined as the embryonal day (E) 0.5. Embryos were collected at E14.5 and E15.5. Females were euthanized and embryos removed and rapidly frozen in cold isopentane (2-Methylbutane, −20°/−25°C) before sectioning. Brains were cryo-sectioned in series [P3, series of eight sections; P12 and adult (8–12 weeks), series of 10 sections; embryos (E14.5, E15.5), series of five sections; *N* = 2–4 mice per stage/genotype/detection].

#### Colorimetric *in situ* Hybridization and Fluorescent *in situ* Hybridization

##### Riboprobes

Detection of TrpV1, Th, Vglut2, Viaat, Dat, NeuroD6, Grp mRNA, and Vmat2 [Probe 2 (*Vmat2 exon 2*) and Probe 6-15 (*Vmat2 exon 6-15*)] mRNA in brain tissue using Colorimetric *In situ* Hybridization (CISH) and/or Fluorescent ISH (FISH) was performed using a previously published protocol (Bimpisidis et al., 2019). Riboprobes detecting the following sequences were prepared: TrpV1: NM_001001445.2; bases 426-1239. Th: NM_009377.1; bases 456-1453. Dat: NM_012694.2; bases 1015-1938. Vglut2: NM_080853.3; bases 2315-3244; Viaat: NM_009508.2; bases 649-1488; NeuroD6: NM_009717.2; bases 632-1420. Grp: NM_175012.4; bases 127-851. Vmat2 Probe 6-15: Vmat2: NM_0130331.1; bases 701-1439 (corresponds to exon 6-15 of mouse sequence NM_172523.3). Vmat2 Probe 2: NM_172523.3; bases 142-274 covering the whole *exon 2* of the *Vmat2* gene. Digoxigenin, fluorescein, and DNP-labeled RNA probes were made by a transcriptional reaction with the incorporation of digoxigenin or fluorescein-labeled nucleotides. The specificity of probes was verified using NCBI blast.

##### Hybridization and Detection

For the hybridization step, coronal cryosections were air-dried, fixed in 4% paraformaldehyde, and acetylated in.25% acetic anhydride/100-mM triethanolamine (pH 8), followed by hybridization for 18 h at 65°C in 100 μl of formamide-buffer containing a 1-μg/ml digoxigenin (DIG)-labeled probe for colorimetric detection or 1-μg/ml DIG-labeled and 1-μg/ml fluorescein-labeled probes for fluorescent detection. Sections were washed at 65°C with SSC buffers of decreasing strength and blocked with 20% FBS and 1% blocking solution. For colorimetric detection, DIG epitopes were detected with alkaline phosphatase-coupled anti-DIG fab fragments at 1/1,000 and a signal developed with NBT/BCIP (*p*-nitroblue tetrazolium chloride/5-bromo-4-chloro-3-indolyl phosphate; 1/100). For fluorescent detection, sections were incubated with horseradish peroxidase (HRP)-conjugated anti-fluorescein antibody (1/5,000). Signals were revealed using Cy2-tyramide (1/250). HRP-activity was stopped by incubation of sections in.1-M glycine, followed by a 3% H_2_O_2_ treatment. DIG epitopes were detected with HRP anti-DIG Fab fragments (1/2,000) and revealed using Cy3 tyramide (1/100). DNP epitopes were detected with HRP anti-DNP Fab fragments (1/2,000) and revealed using Cy3 tyramide (1/100). Nuclear staining was performed with 4’ 6-diamidino-2-phenylindole (DAPI). All slides were scanned on a NanoZoomer 2.0-HT (Hamamatsu Photonics, Hamamatsu City, Japan) at 20x resolution. Laser intensity and time of acquisition were set separately for each riboprobe. Images were analyzed using the NDP.view2 software (Hamamatsu Photonics). Published atlases (Franklin and Paxinos, 2013) were used to outline anatomical borders.

##### Definition of Positive Cells and Counting

For a cell to be considered as TrpV1-positive (TrpV1^+^), fluorescent TrpV1 mRNA labeling was defined as a cluster of fluorescent dots (minimum six dots) on the cell surface in agreement with the cellular colorimetric TrpV1 labeling. The background was defined by a single dot, with no clustering. The fluorescent stain 4′,6-diamidino-2-phenylindole (DAPI) was used to define cell nuclei. Since TrpV1 mRNA labeling was generally weak (defined as weaker than labeling for Th and Vglut2 in the same area), criteria for cell counting were based on three requirements: Size of cells strongly positive for TrpV1; the size of Th and Vglut2 positive cells in regions where TrpV1 is strongly expressed; one cell nucleus present. Based on these criteria, a circle of diameter of 11 μm was generated for each such defined TrpV1^+^ cell. Manual counting of TrpV1^+^ cells was performed in CISH and FISH detections. Counting were made in all regions in which Trpv1 mRNA was detected: VTA (subnuclei IF, PN, PBP, RLi, and CLi), PHA, and paraventricular nucleus, spanning Bregma −2.18 to Bregma −4.16 (Franklin and Paxinos, 2013). To define an area, outlines defined by CISH detection were used (see Supplementary Figure 1 as an example of outlines defined in a CISH detection). Counting was performed in both CISH and FISH detections (*N* = 3 mice per detection and probe combination, serial sections). Similar results obtained using both detections validated the method.

##### Co-fluorescent *in situ* Hybridization Analysis

The percentage of TrpV1^+^ cells co-labeled with each marker (TrpV1/Th, TrpV1/Vmat2, Trpv1/Vglut2, TrpV1/Dat, Trpv1/Viaat, Trpv1/Grp, and TrpV1/NeuroD6) was established in each VTA subnucleus using CISH detection to define outlines as described above.

##### Vmat2 Wild Type and Conditional Knockout Cells

*TrpV1^*Cre*+/wt^;Vmat2^*flox/flox*^TrpV1^*Cre–/wt*^;Vmat2^*flox/flox*^* mice at E15.5 and adult stages (*N* = 3 mice per genotype and stage; serial sections per probe combination) were analyzed using a probe mixture of Vmat2 Probe 2 and Vmat2 Probe 6–15. The occurrence of cells positive for both probes (representing *Vmat2* wild-type cells) and cells positive for Probe 6-15 only (*Vmat2*cKO cells) was quantified by manual counting, using detection of Vmat2 Probe 6–15 and the Thprobe as references for anatomical boundaries and outline of distinct cell soma.

### Stereotaxic Virus Injection

Stereotaxic injections were performed on anesthetized *TrpV1^*Cre*+/wt^* mice (9–12 weeks of age; *N* = 11 male and female mice) maintained at 1.4–1.8% isoflurane-air mix v/v (0.5–2 L/min). Prior to surgery and, also, 24-h post-surgery, mice received a subcutaneous injection of analgesics (Carprofen; 5-mg/kg, Norocarp). A topical analgesic (Marcain; 1.5 mg/kg, AstraZeneca) was locally injected on the site of the incision. After exposing the skull, drill holes were prepared. The mice were unilaterally injected in the PHA/VTA region with an adeno-associated (AAV) virus containing a floxed DNA construct carrying the gene encoding the yellow fluorescent protein, eYFP (rAAV2/EF1a-DIO-eYFP). Virus concentration was 4.6 × 10^12^ virus molecules/ml delivered at the following mouse brain coordinate (Franklin and Paxinos, 2013): anteroposterior (AP) = −2.80 mm, mediolateral (ML) = −0.60 mm from the midline with an 8° angle in the frontal plan to avoid the sagittal vein, dorsoventral level (DV) = −4.40 mm from the dura matter. About 300 nL of virus solution was injected with a NanoFil syringe (World Precision Instruments, Sarasota, FL, United States) at the speed of 100 nL per minute. The rAAV2/EF1a-DIO-eYFP was purchased from UNC Vector Core, Chapel Hill, NC, USA, in accordance with Material Transfer Agreement. Injected mice are referred to as *TrpV1::EF1a-DIO-eYFP* mice.

### Immunohistochemistry Analysis

#### Fluorescent Immunohistochemistry

*TrpV1::EF1a-DIO-eYFP* mice (13–18-week old mice: 4–6 weeks after injection at 9–12 weeks of age) were deeply anesthetized and perfused trans-cardially with phosphate-buffer-saline (PBS), followed by ice-cold 4% formaldehyde. Brains were extracted and 60-μm vibratome-cut sections were freshly prepared. Fluorescent immunohistochemistry was performed to detect TH and enhance the YFP signal. After rinsing in PBS, sections were incubated for 90 min in PBS.3% X-100 Triton, containing 5% blocking solution (normal donkey serum), followed by incubation with a primary antibody (Rabbit anti-TH, ab152, Millipore, 1/1,000; chicken anti-GFP 1/1,000, cat. No. ab13970, Abcam), diluted in 1% normal donkey serum in PBS, overnight at 4°C. The next day, sections were rinsed in PBS plus.1% Tween-20 solution and incubated for 90 min with a secondary antibody diluted in PBS (A488 donkey anti-chicken 1/1,000, cat. No. 703-545-155, Jackson ImmunoResearch). After rinsing in PBS containing.1% Tween-20, sections were incubated for 30 min with DAPI diluted in distilled water (1/5,000). Sections were mounted with a Fluoromount aqueous mounting medium (Sigma, United States) and cover-slipped. Sections were digitally imaged with the NanoZoomer 2-0-HT.0 scanner (Hamamatsu) and visualized with NDPView2 software (Hamamatsu). YFP-positive cell bodies, fibers, and projections were analyzed and evaluated upon comparison with TH (visualizing brain monoamine systems) and DAPI staining.

#### Chromogenic Immunohistochemistry

*Trpv1^*Cre*–/wt^;Vmat2^*flox/flox*^* control (*N* = 4; three males, one female) and *Trpv1^*Cre*+/wt^;Vmat2^*flox/flox*^*cKO (*N* = 4; three males, one female) mice (24 weeks of age) were perfused trans-cardially with PBS, followed by ice-cold 4% formaldehyde. Brains were extracted, cryoprotected in 30% sucrose solution, and stored at −80°C until sectioning at 60-μm sections with a cryostat. Sections were washed with 1% H_2_O_2_ in PBS with.1% Triton-X for endogenous peroxidase activity inhibition and incubated for 90 min with a blocking solution containing 5% goat serum. Sections were then incubated with primary TH antibody (Rabbit anti-TH, ab152, Millipore, 1/4,000) overnight at 4°C, followed by 90-min incubation with a biotinylated anti-rabbit antibody and subsequent 90-min incubation with ABC solution (Vectastain ABC kit). Sections were washed with.1-M a Tris-HCl buffer (pH 7.4) and exposed to DAB solution (a DAB peroxidase substrate kit, Vector Laboratories) until a signal appeared. The chromogenic reaction was then blocked with Tris buffer. Sections were counterstained with cresyl violet followed by dehydration with increasing ethanol concentrations (75, 90, and 100%) and histological clearing using Histoclear (Histolab). Sections were mounted on slides using a DPX mounting medium (Sigma) and cover-slipped. Sections were digitally imaged with the NanoZoomer 2-0-HT.0 (Hamamatsu) scanner and visualized with NDPView2 software (Hamamatsu). TH-positive cell bodies, fibers, and projections were analyzed and evaluated by comparison between genotypes.

### Behavior Analysis

*TrpV1^*Cre*+/wt^;Vmat2^*flox/flox*^*cKO and *TrpV1^*Cre*–/wt^;Vmat2^*flox/flox*^* control (Ctrl) littermate mice were analyzed in behavior experiments. Throughout the process, mice had access to food and water *ad libitum* in standard humidity and temperature conditions and with a 12-h dark/light cycle. Behavioral tests were performed during the light cycle between 9:00 a.m. and 4:00 p.m. All mice were handled for 4 days prior to behavioral testing and habituated to the experimental room for 30 min before handling and testing. Young adult (YA) mice were 8 weeks old, and mature adult (MA) mice were 18 weeks old at the start of the behavioral testing. Each mouse performed either the open field test or the elevated plus maze (both of which consist of a 1-day test) 24 h prior to starting the amphetamine sensitization protocol. Mice (males and females mixed) were analyzed in the following behavioral tests according to the procedure described below:

#### Open Field Test

Mice (Ctrl/YA N = 6, two males, four females; cKO/YA N = 9, five males, four females; Ctrl/MAN = 7, three males, four females; cKO/MAN = 6, three males, three females) were individually placed in the central zone of the open field arena and allowed to freely explore it for 10 min. The open-field chamber consisted of a 50-cm, squared, transparent, plastic arena with a white floor that has been divided into a central zone (center, 25% of the total area) and a peripheral zone (borders). Total distance moved, time spent, and frequency in crossing to the center, time spent in the corners, time spent not moving, and body elongation were automatically documented. Rearing, self-grooming, and sniffing behaviors were manually recorded by an experimenter blind to the experimental groups using the EthoVision XT tracking software (Noldus Information Technology, Netherlands).

#### Elevated Plus Maze

The elevated plus maze apparatus consists of two open arms (35-cm length) and two closed arms (35-cm length) in which walls (15-cm high) provide shelter; the open and closed arms cross in the middle to create a center platform. The maze is elevated 50 cm from the floor. Mice (Ctrl/YAN = 8, two males, six females; cKO/YA *N* = 13, six males, seven females; Ctrl/MA N = 11, six males, five females; cKO/MA *N* = 12, five males, seven females) were placed individually in the center of the maze facing one of the open arms and allowed to freely explore the apparatus for 10 min. The results of the test were recorded with a camera placed above the EPM arena. Time spent in arms, number of entries in arms, number of head dips, distance moved, time spent moving and body elongation were automatically scored by the EthovisionXT software (Noldus Information Technology, The Netherlands).

#### Baseline Locomotion

Spontaneous locomotion was monitored for 30 min upon placing the mice (Ctrl/YA *N* = 14, 4 males, 10 females; cKO/YA N = 22, 11 males, 11 females; Ctrl/MA *N* = 18, nine males, nine females; cKO/MAN = 18, 8 males, 10 females) in Makrolon polycarbonate boxes covered with a transparent Plexiglas lid, containing 1.5-cm bedding. Locomotion was recorded by the EthovisionXT software (Noldus Information Technology, Netherlands).

#### Amphetamine Sensitization

Mice (Ctrl/YA *N* = 14, 4 males, 10 females; cKO/YA *N* = 22, 11 males, 11 females; Ctrl/MA *N* = 18, nine males, nine females; cKO/MAN = 18, 8 males, 10 females) received a saline injection (Day 1), followed by four consecutive days of amphetamine injection (Days 2–5, 3 mg/kg, i.p.), followed by a last injection on Day 17 (3 mg/kg, i.p.). Locomotion was recorded 30 min before (baseline) and 90 min after injection using the EthovisionXT software (Noldus Information Technology, Netherlands). The software allowed the calculation for the entire period (90 min) as well as measures of different sub-periods (10 min). Distance moved across 90 min represents the sum of 10-min sub-periods.

#### Statistical Analysis

All mice used for the behavioral studies were included in the analysis.

Repeated measures (RM) two-way ANOVA with Greenhouse–Geisser corrections were used to compare the mean of the weight of control and cKO mice. *Post-hoc* comparisons were performed by Sidak’s multiple comparison test.

Unpaired *t*-tests were used to compare the mean scores of control and cKO mice in the open field test.

Repeated measures two-way ANOVA was used to compare the mean of scores for time spent and the number of visits in arms for the EPM. *Post hoc* comparisons were performed by Sidak’s multiple comparison test. Unpaired *t*-tests were used to compare mean scores of control and cKO mice for other parameters of the elevated plus maze.

Repeated measures two-way ANOVA was used to compare the mean of baseline locomotion. *Post hoc* comparisons were performed by Sidak’s multiple comparison test.

Repeated measures one-way ANOVA with Greenhouse–Geisser corrections were used to compare mean scores of amphetamine-induced locomotion across a session for every single group. *Post hoc* comparisons were performed by Dunnett’s multiple comparison tests to compare Day 2 with other sessions.

Repeated measures two-way ANOVA with Greenhouse–Geisser corrections were used to compare mean scores of amphetamine-induced locomotion. *Post hoc* comparisons were performed by Sidak’s multiple comparison test.

Data are presented as mean ± SEM. Data analysis was performed with Prism (GraphPad Prism version 9.00 for Windows, GraphPad Software, La Jolla, CA, United States).

Details from the statistical analysis are available in Supplementary File 1.

## Significance Statement

Teasing out the impact of distinct brain neurons on behavioral regulation is critical in neuroscience. TRPV1 is well known for its role in heat and pain processing *via* peripheral sensory neurons. However, the distribution of this receptor in the brain has remained elusive. This study identifies a peak of TrpV1 mRNA in the ventral tegmental area (VTA) of the mouse midbrain at the perinatal stage, allowing for its careful histological characterization. TrpV1 is primarily detected in medial VTA subnuclei but is absent from the substantia nigra *pars compacta* (SNc). The far majority of TrpV1 mRNA co-localizes with markers of dopamine (Th, Vmat2) and glutamate (Vglut2) neurons. This TrpV1^+^/Th^+^/Vglut2^+^/Vmat2^+^ molecular signature thus defines a distinct subpopulation within the dopamine-glutamate (DA-GLU) co-releasing neuronal population present within the VTA. In accordance with a role for such DA-GLU neurons in psychostimulant response, selective manipulation of dopamine release by this TrpV1^+^ subpopulation was sufficient to modulate amphetamine-induced psychomotor behavior. This study highlights the behavioral role of a distinct group of VTA neurons.

## Data Availability Statement

The raw data supporting the conclusions of this article will be made available by the authors, without undue reservation.

## Ethics Statement

The animal study outline was reviewed and approved by Uppsala Local Committee.

## Author Contributions

GS: behavior experiments, data analysis, figure preparation, and manuscript text (original and revision). AG: tracing and immunohistochemistry, data analysis, manuscript revision, and figure preparation. SD: *in situ* hybridization, data analysis, figure preparation, and manuscript revision. BV: tracing and immunohistochemistry, data analysis, figure preparation, and manuscript revision. ÅW-M: project design and funding, data analysis, figure preparation, and manuscript text writing (original draft, editing, and revision).

## Conflict of Interest

SD is the owner of Oramacell, Paris. The remianing authors declare that the research was conducted in the absence of any commercial or financial relationships that could be construed as a potential conflict of interest.

## Publisher’s Note

All claims expressed in this article are solely those of the authors and do not necessarily represent those of their affiliated organizations, or those of the publisher, the editors and the reviewers. Any product that may be evaluated in this article, or claim that may be made by its manufacturer, is not guaranteed or endorsed by the publisher.

## Supplementary Material

Supplementary Figure 1 Colorimetric and fluorescent in situ hybridization (CISH and FISH) of coronal mouse brain sections at postnatal day (P) **3. (A,B)** The area encompassing the posterior hypothalamus (including PHA, RM) displayed. DAPI is used for the detection of cell nuclei. Top panel, CISH: TrpV1 **(A1)**, Th **(A2)**, Vglut2 **(A3)**. Bottom panel, FISH: TrpV1/Th **(B1)**, TrpV1/Vglut2 **(B2)**, TrpV1/Viaat **(B3)**; positive cells indicated in insets by arrows; white arrows indicate co-labeling of red and green fluorophores. **(C)** Bottom: TrpV1/Viaat **(C)**, the same section level as shown in Figure 1A. Scale bars, 500 μm **(A)**; 120 μm (**B**, insets 20 μm); 100 μm (**C**, insets 10, μm). IF, interfascicular nucleus; PBP parabrachial pigmented nucleus; PHA, posterior hypothalamic nucleus; PN, paranigral nucleus; RM, retromammillary nucleus; RLi, rostral linear nucleus; SNc, substantia nigra *pars compacta*; VTA, ventral tegmental area.

Supplementary Figure 2 Colorimetricribo probe in situ hybridization of serial sections at mouse embryonic Day (E) 14.5, postnatal day (P) P12, and adult (9 weeks). The area encompassing the posterior hypothalamus (including PHA and RM) and midbrain (including VTA and SNc) was displayed. Detection of TrpV1, Th, Vglut2 mRNAs in **(A)** sagittal and horizontal sections at E14.5 [a line in **A1,A3,A5** indicates section level (S1) shown in **A2,A4,A6**]; **(B)** coronal sections at P12; **(C)** coronal sections of the adult mouse brain. Scale bars, 500 μm. ms, mesencephalic flexure; RLi, rostral linear nucleus; IF, interfascicular nucleus; PBP, parabrachial pigmented nucleus; PHA, posterior hypothalamic nucleus; PN, paranigral nucleus; RM, retromammillary nucleus; SNc, substantia nigra *pars compacta*; VTA, ventral tegmental area.

Supplementary Figure 3 Double-labeling riboprobe fluorescent in situ hybridization (FISH) of serial brain sections at postnatal day (P) 3 identifying TrpV1 co-localization with Vmat2 in VTA subnuclei. Left panel, TrpV1 (red), Vmat2 (green); sections encompassing the posterior hypothalamus through the VTA toward, and including, the CLi and A8. Yellow indicates co-localization TrpV1/Vmat2. DAPI is used for the detection of cell nuclei. Right-side panels show close-ups of cells in areas indicated by a square in the left panel. Positive cells are indicated in insets by arrowheads; white arrows indicate co-labeling of red and green fluorophores. Scale bars, 500 μm (12 μm in insets where each dotted line indicates a discrete cell). RLi, rostral linear nucleus; IF, interfascicular nucleus; PN, paranigral nucleus; PBP, parabrachial pigmented nucleus; PHA, posterior hypothalamic nucleus; RM, retromammillary nucleus; PBP, parabrachial pigmented nucleus; SNc, substantia nigra *pars compacta*; CLi, caudal linear nucleus; A8, A8 dopamine area; VTA, ventral tegmental area.

Supplementary Figure 4 Colorimetric riboprobe in situ hybridization (CISH) of serial brain sections at postnatal day (P) 3. TrpV1, Th, NeuroD6, Grp mRNAs analyzed in coronal sections encompassing the posterior hypothalamus through the VTA toward, and including, the CLi and A8. TrpV1 **(A1,A5,A9,A13)**; Th **(A2,A6,A10,A14)**; NeuroD6 **(A3,A7,A11,A15)**; Grp **(A4,A8,A12,A16)**. Scale bars, 500 μm. A8, A8 dopaminearea; CLi, caudal linear nucleus; IF, interfascicular nucleus; PN, paranigral nucleus; PBP, parabrachial pigmented nucleus; PHA, posterior hypothalamic nucleus; RM, retromammillary nucleus; RLi, rostral linear nucleus; SNc, substantia nigra *pars compacta*; VTA, ventral tegmental area; R, rostral; C, caudal.

Supplementary Figure 5 Vmat2 riboprobe in situ hybridization. Serial sections throughout the VTA area in *TrpV1^*Cre*+/wt^;Vmat2^*flox/flox*^* conditional knockout (cKO) mice (12 weeks old). DAPI is used for the detection of cell nuclei. **(A)** Triple fluorescent *in situ* hybridization (FISH) co-assessing Th mRNA with Vmat2 Probe 2 and Vmat2 Probe 6–15 using the two-probe approach illustrated in Figure 5 combined with the Th probe. Scale bar, 500 μm. **(B)** Mid-sagittal brain sections of a mouse embryo at E15.5 (covering the area around the mesencephalic flexure where midbrain dopamine neurons are born), showing labeling of Vmat2 Probe 2 (purple) and Probe 6–15 (green). A square in top panels indicates an area selected for a closeup in bottom panels. Only few cells show the presence of only Probe 6–15 (an indicator of the cKO phenotype); most cells are positive for both Vmat2 probes at this stage. Scale bar, 300 μm. CLi, caudal linear nucleus; IF, interfascicular nucleus; PN, paranigral nucleus; PBP, parabrachial pigmented nucleus; RLi, rostral linear nucleus; SNc, substantia *nigra pars compacta*; VTA, ventral tegmental area.

Supplementary Figure 6 Weight analysis. Analysis and comparison of *TrpV1^*Cre–/wt*^;Vmat2^*flox/flox*^* control (Ctrl) and *TrpV1^*Cre*+/wt^;Vmat2^*flox/flox*^* conditional knockout (cKO) mice. **(A)** A weight curve for Ctrl (*N* = 31) and cKO (*N* = 25) mice. Weight is expressed in grams for each week ± SEM (###*p* < 0.001, age effect).

Supplementary Figure 7 Open-field and elevated-plus maze paradigms. Analysis and comparison of *TrpV1^*Cre–/wt*^;Vmat2^*flox/flox*^* control (Ctrl) and *TrpV1^*Cre*+/wt^;Vmat2^*flox/flox*^* conditional knockout (cKO) mice at young adult (YA; 8 weeks old) and mature adult (MA; 18 weeks old) age in the open field and plus-maze paradigms. Open field test (OFT), Ctrl/YA (*N* = 6) and cKO/YA (*N* = 9), Ctrl/MA (*N* = 7) and cKO/MA (*N* = 6) data are expressed as mean ± SEM. **(A)** Time spent not moving YA. **(B)** Self-grooming YA. **(C)** Visits to the center YA. **(D)** Time spent in corners YA. **(E)** Time spent contracted YA. **(F)** Time spent not moving MA. **(G)** Self-grooming MA. **(H)** Visits to the center MA. **(I)** Time spent in corners MA. **(J)** Time spent contracted MA. Elevated plus maze (EPM), Ctrl/YA (*N* = 8) and cKO/YA (*N* = 13), Ctrl/MA (*N* = 11) and cKO/MA (*N* = 12), data are expressed as mean ± SEM. **(K)** Distance moved YA. **(L)** Time spent moving YA. **(M)** Number of head dips YA. **(N)** Time spent stretched YA. **(O)** Distance moved MA. **(P)** Time spent moving MA. **(Q)** Number of head dips MA. **(R)** Time spent stretched MA.

Supplementary Figure 8 Amphetamine-induced locomotion. Analysis and comparison of *TrpV1^*Cre–/wt*^;Vmat2^*flox/flox*^* control (Ctrl) and *TrpV1^*Cre*+/wt^;Vmat2^*flox/flox*^* conditional knockout (cKO) mice at young adult (YA; 8 weeks old) and mature adult (MA; 18 weeks old) age in the amphetamine sensitization paradigm. Amphetamine-induced locomotion, 90 min following amphetamine injection (YA); Ctrl/YA (*N* = 22) and cKO/YA mice (*N* = 22). **(A)** Amphetamine-induced locomotion on Day 4. Distance moved presented as mean ± SEM for each 10-min period (###*p* < 0.001, time effect; +*p* = 0.023, time × genotype interaction). **(B)** Amphetamine-induced locomotion on Day 5. Distance moved presented as mean ± SEM for each 10-min period (###*p* < 0.001, time effect). **(C)** Amphetamine-induced locomotion on Day 17. Distance moved presented as mean ± SEM for each 10-min period (###*p* < .001, time effect; +*p* = 0.039, time × genotype interaction). Amphetamine-induced locomotion, 90 min following amphetamine injection (MA); Ctrl/MA (*N* = 18) and cKO/MA mice (*N* = 18). **(D)** Amphetamine-induced locomotion on Day 4. Distance moved presented as mean ± SEM for each 10-min period (###*p* < 0.001, time effect). **(E)** Amphetamine-induced locomotion on Day 5. Distance moved presented as mean ± SEM for each 10-min period [###*p* < 0.001, time effect; +*p* = 0.02, time × genotype interaction)]. **(F)** Amphetamine-induced locomotion on Day 17. Distance moved presented as mean ± SEM for each 10-min period (###*p* < 0.001, time effect; ++*p* = 0.008, time × genotype interaction).

Supplementary Table 1 The extent of cells detected as positive for targeted (cKO) Vmat2 mRNA. The table shows the percentage of Vmat2 cKO cells in several brain areas as indicated by the terminology (including the hypothalamus, mammillary nuclei, and monoaminergic neuronal populations of the VTA, SNc, raphe, and medulla) and distribution of Vmat2 cKO cells within the VTA. Analysis performed in adult mice (12 weeks old). *N* = 3 mice, serial sections per detection. RLi, rostral linear nucleus; IF, interfascicular nucleus; PIF, parainterfascicular nucleus; PN, paranigral nucleus; PHA, posterior hypothalamic nucleus; RM, retromammillary nucleus; PBP, parabrachial pigmented nucleus; SNc, substantia nigra *pars compacta*; CLi, caudal linear nucleus; A8, A8 dopamine area (aka RRF, retrorubral field); VTA, ventral tegmental area; LC, locus coeruleus; C1–C3, adrenergic C1–C3 cell groups; Arc, arcuate nucleus; LH, lateral hypothalamus; DR, dorsal raphe; A11, A11 dopamine area; AH, anterior hypothalamus nucleus; PMV, premammillary nucleus ventral part; MnR, median raphe nucleus; DRI, dorsal raphe interfascicular part; RMg, raphe magnus nucleus; RIP, raphe interpositus nucleus; R0b, raphe obscurus nucleus; LPGi, lateral paragigantocellular nucleus.

Supplementary File 1 Statistical analysis of results obtained in behavior analysis. *TrpV1^*Cre–/wt*^;Vmat2^*flox/flox*^*control (Ctrl) and *TrpV1^*Cre*+/wt^;Vmat2^*flox/flox*^* conditional knockout (cKO) mice at young adult (YA; 8 weeks old) and mature adult (MA; 18 weeks old) age were analyzed in behavioral paradigms. The file shows the detailed statistical analysis and results obtained from these experiments, including the number of animals and sex for each test, with reference to the figure where data are displayed.
