# Supplementary figures and images for: Midbrain Dopamine Neurons Defined by TrpV1 Modulate Psychomotor Behavior
Source: Front Neural Circuits. 2021 Nov 11;15:726893. doi: 10.3389/fncir.2021.726893 (PMC8632262; doi:10.3389/fncir.2021.726893)

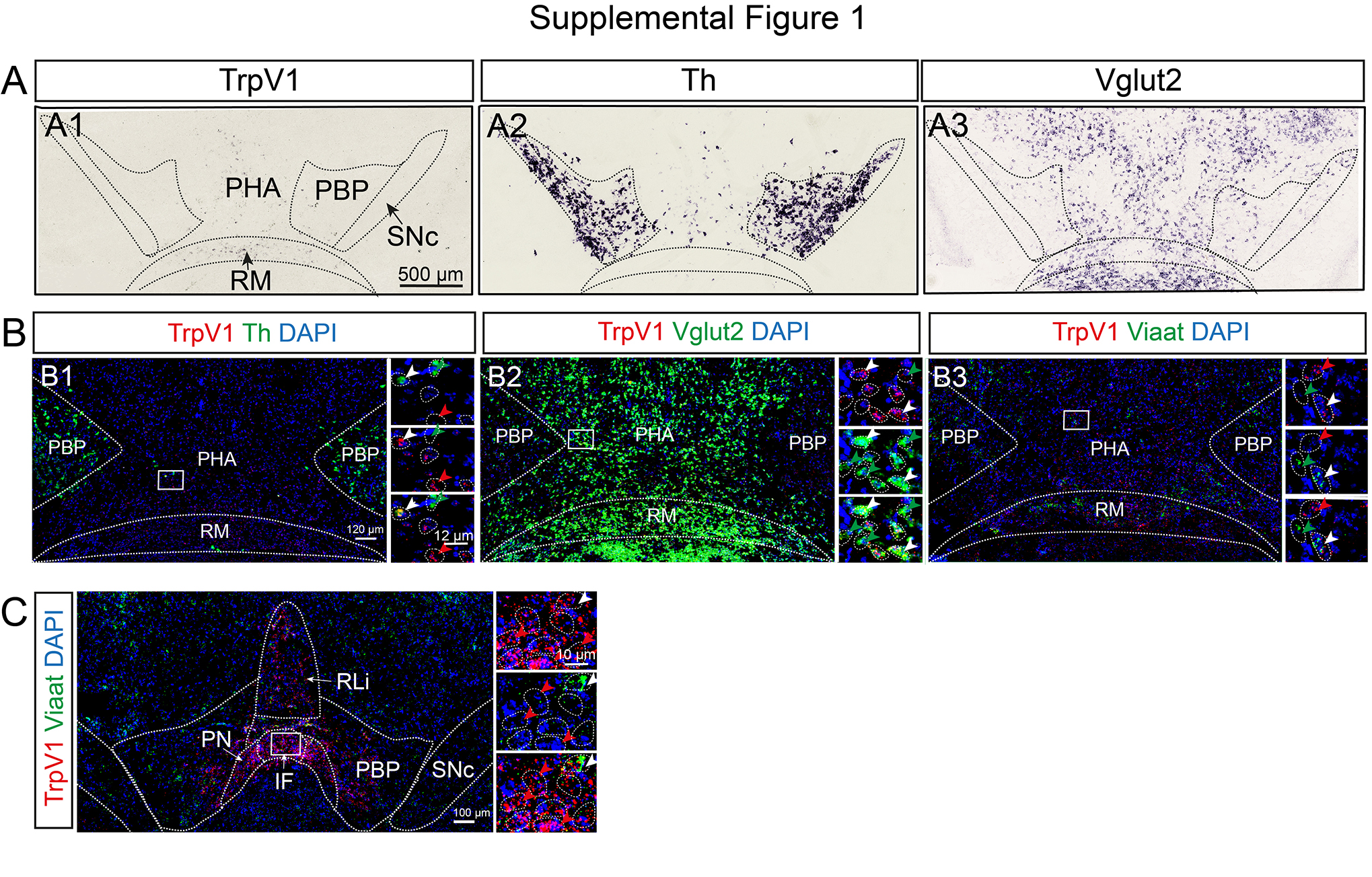

Supplement: Supplementary Figure 1 — Colorimetric and fluorescent in situ hybridization (CISH and FISH) of coronal mouse brain sections at postnatal day (P) 3. (A,B) The area encompassing the posterior hypothalamus (including PHA, RM) displayed. DAPI is used for the detection of cell nuclei. Top panel, CISH: TrpV1 (A1), Th (A2), Vglut2 (A3). Bottom panel, FISH: TrpV1/Th (B1), TrpV1/Vglut2 (B2), TrpV1/Viaat (B3); positive cells indicated in insets by arrows; white arrows indicate co-labeling of red and green fluorophores. (C) Bottom: TrpV1/Viaat (C), the same section level as shown in Figure 1A. Scale bars, 500 μm (A); 120 μm (B, insets 20 μm); 100 μm (C, insets 10, μm). IF, interfascicular nucleus; PBP parabrachial pigmented nucleus; PHA, posterior hypothalamic nucleus; PN, paranigral nucleus; RM, retromammillary nucleus; RLi, rostral linear nucleus; SNc, substantia nigra pars compacta; VTA, ventral tegmental area. [file Image_1.JPEG]

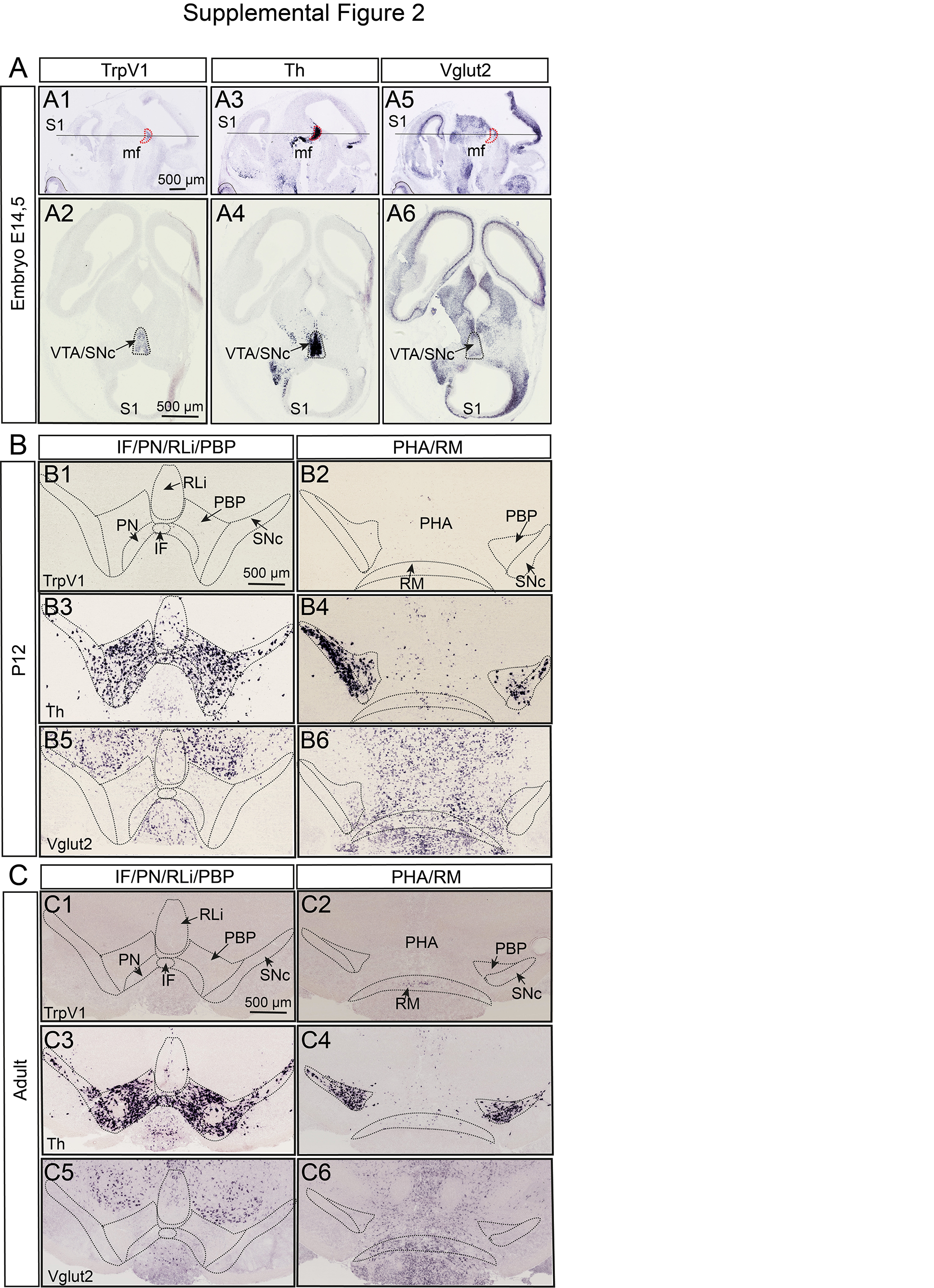

Supplement: Supplementary Figure 2 — Colorimetricribo probe in situ hybridization of serial sections at mouse embryonic Day (E) 14.5, postnatal day (P) P12, and adult (9 weeks). The area encompassing the posterior hypothalamus (including PHA and RM) and midbrain (including VTA and SNc) was displayed. Detection of TrpV1, Th, Vglut2 mRNAs in (A) sagittal and horizontal sections at E14.5 [a line in A1,A3,A5 indicates section level (S1) shown in A2,A4,A6]; (B) coronal sections at P12; (C) coronal sections of the adult mouse brain. Scale bars, 500 μm. ms, mesencephalic flexure; RLi, rostral linear nucleus; IF, interfascicular nucleus; PBP, parabrachial pigmented nucleus; PHA, posterior hypothalamic nucleus; PN, paranigral nucleus; RM, retromammillary nucleus; SNc, substantia nigra pars compacta; VTA, ventral tegmental area. [file Image_2.JPEG]

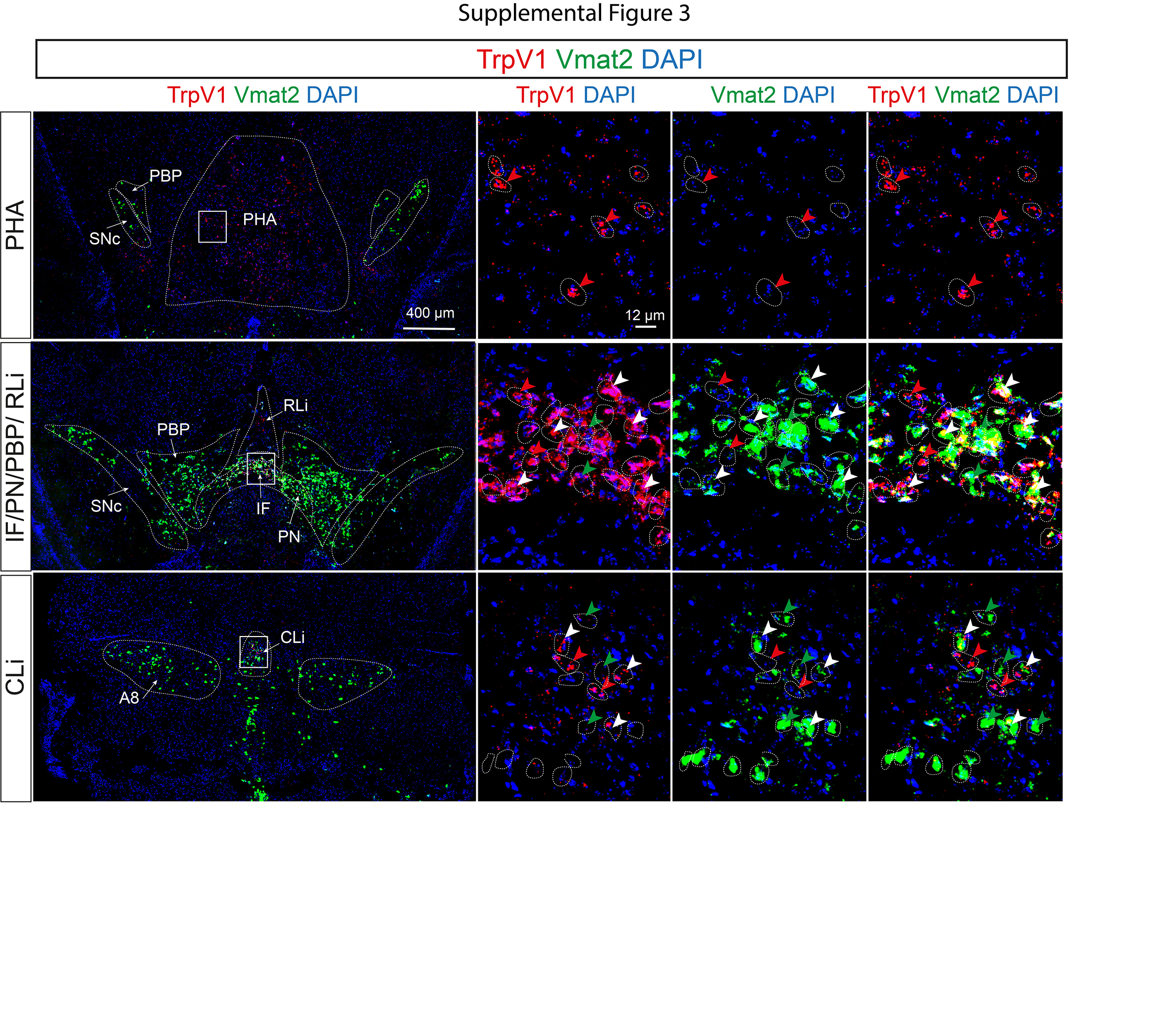

Supplement: Supplementary Figure 3 — Double-labeling riboprobe fluorescent in situ hybridization (FISH) of serial brain sections at postnatal day (P) 3 identifying TrpV1 co-localization with Vmat2 in VTA subnuclei. Left panel, TrpV1 (red), Vmat2 (green); sections encompassing the posterior hypothalamus through the VTA toward, and including, the CLi and A8. Yellow indicates co-localization TrpV1/Vmat2. DAPI is used for the detection of cell nuclei. Right-side panels show close-ups of cells in areas indicated by a square in the left panel. Positive cells are indicated in insets by arrowheads; white arrows indicate co-labeling of red and green fluorophores. Scale bars, 500 μm (12 μm in insets where each dotted line indicates a discrete cell). RLi, rostral linear nucleus; IF, interfascicular nucleus; PN, paranigral nucleus; PBP, parabrachial pigmented nucleus; PHA, posterior hypothalamic nucleus; RM, retromammillary nucleus; PBP, parabrachial pigmented nucleus; SNc, substantia nigra pars compacta; CLi, caudal linear nucleus; A8, A8 dopamine area; VTA, ventral tegmental area. [file Image_3.JPEG]

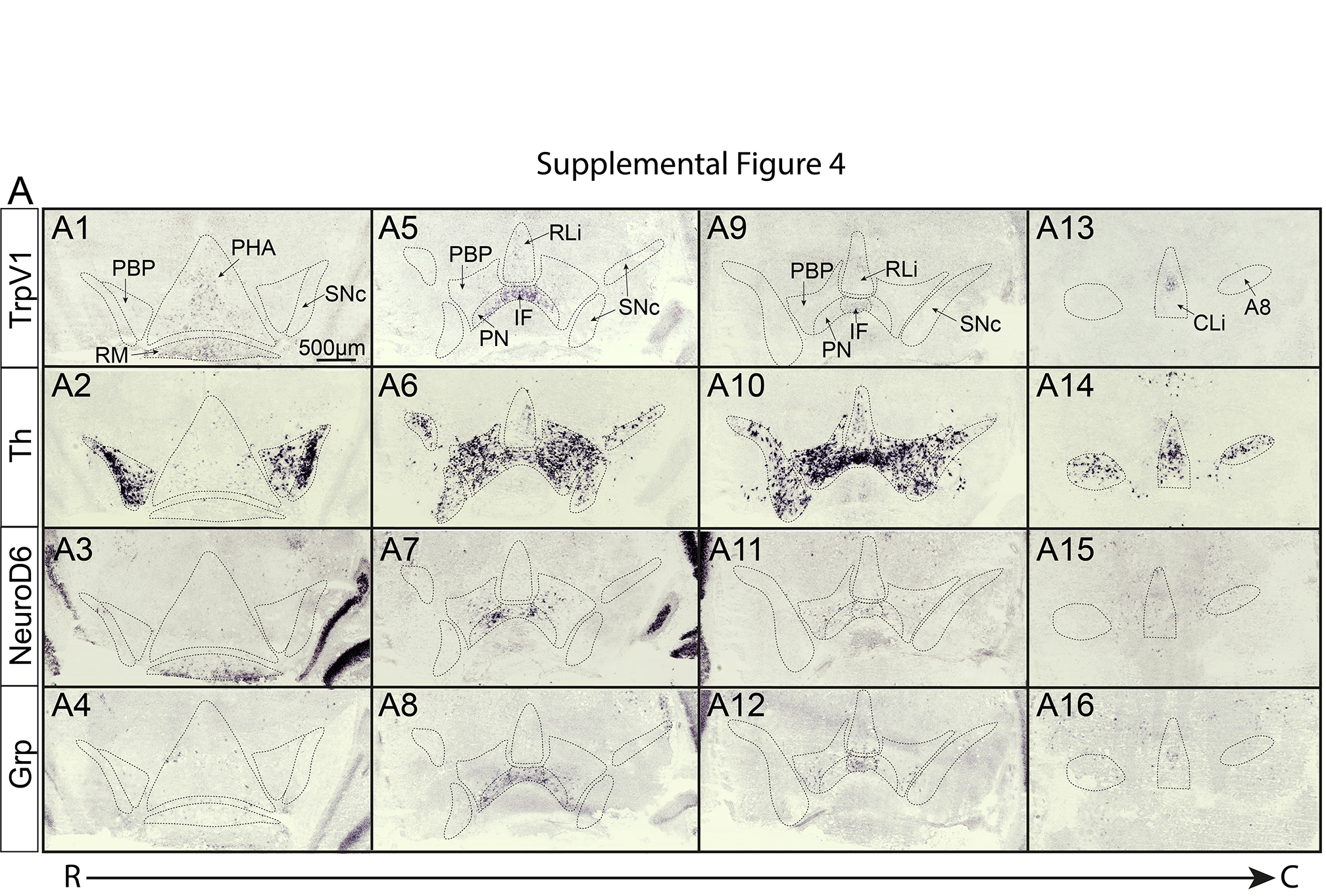

Supplement: Supplementary Figure 4 — Colorimetric riboprobe in situ hybridization (CISH) of serial brain sections at postnatal day (P) 3. TrpV1, Th, NeuroD6, Grp mRNAs analyzed in coronal sections encompassing the posterior hypothalamus through the VTA toward, and including, the CLi and A8. TrpV1 (A1,A5,A9,A13); Th (A2,A6,A10,A14); NeuroD6 (A3,A7,A11,A15); Grp (A4,A8,A12,A16). Scale bars, 500 μm. A8, A8 dopaminearea; CLi, caudal linear nucleus; IF, interfascicular nucleus; PN, paranigral nucleus; PBP, parabrachial pigmented nucleus; PHA, posterior hypothalamic nucleus; RM, retromammillary nucleus; RLi, rostral linear nucleus; SNc, substantia nigra pars compacta; VTA, ventral tegmental area; R, rostral; C, caudal. [file Image_4.JPEG]

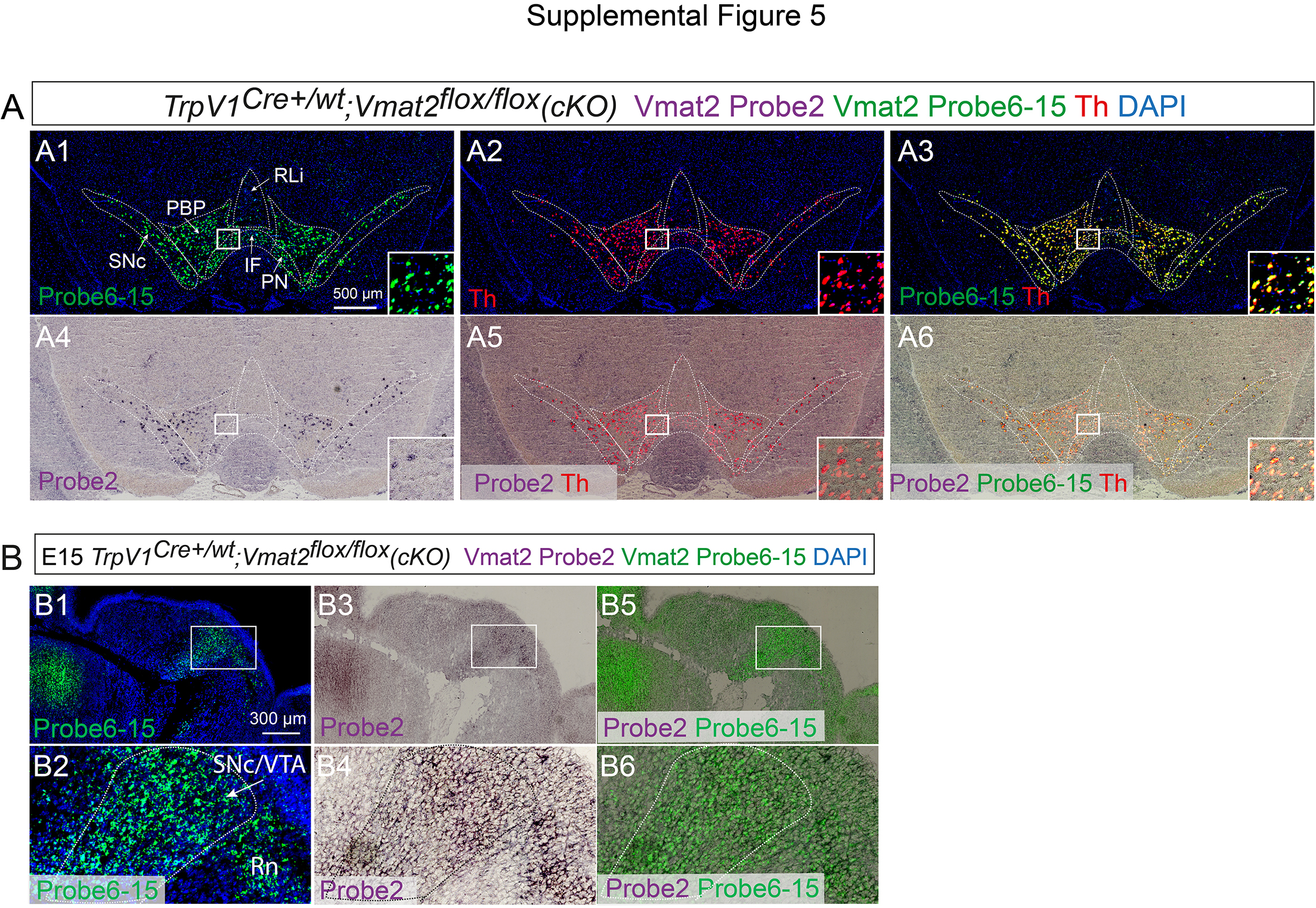

Supplement: Supplementary Figure 5 — Vmat2 riboprobe in situ hybridization. Serial sections throughout the VTA area in TrpV1Cre+/wt;Vmat2flox/flox conditional knockout (cKO) mice (12 weeks old). DAPI is used for the detection of cell nuclei. (A) Triple fluorescent in situ hybridization (FISH) co-assessing Th mRNA with Vmat2 Probe 2 and Vmat2 Probe 6–15 using the two-probe approach illustrated in Figure 5 combined with the Th probe. Scale bar, 500 μm. (B) Mid-sagittal brain sections of a mouse embryo at E15.5 (covering the area around the mesencephalic flexure where midbrain dopamine neurons are born), showing labeling of Vmat2 Probe 2 (purple) and Probe 6–15 (green). A square in top panels indicates an area selected for a closeup in bottom panels. Only few cells show the presence of only Probe 6–15 (an indicator of the cKO phenotype); most cells are positive for both Vmat2 probes at this stage. Scale bar, 300 μm. CLi, caudal linear nucleus; IF, interfascicular nucleus; PN, paranigral nucleus; PBP, parabrachial pigmented nucleus; RLi, rostral linear nucleus; SNc, substantia nigra pars compacta; VTA, ventral tegmental area. [file Image_5.JPEG]

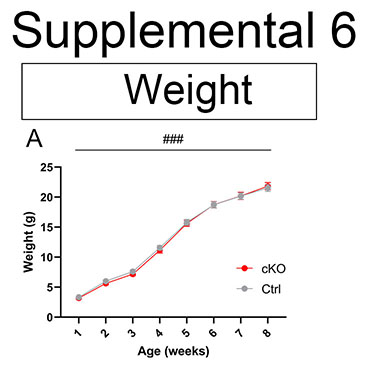

Supplement: Supplementary Figure 6 — Weight analysis. Analysis and comparison of TrpV1Cre–/wt;Vmat2flox/flox control (Ctrl) and TrpV1Cre+/wt;Vmat2flox/flox conditional knockout (cKO) mice. (A) A weight curve for Ctrl (N = 31) and cKO (N = 25) mice. Weight is expressed in grams for each week ± SEM (###p < 0.001, age effect). [file Image_6.JPEG]

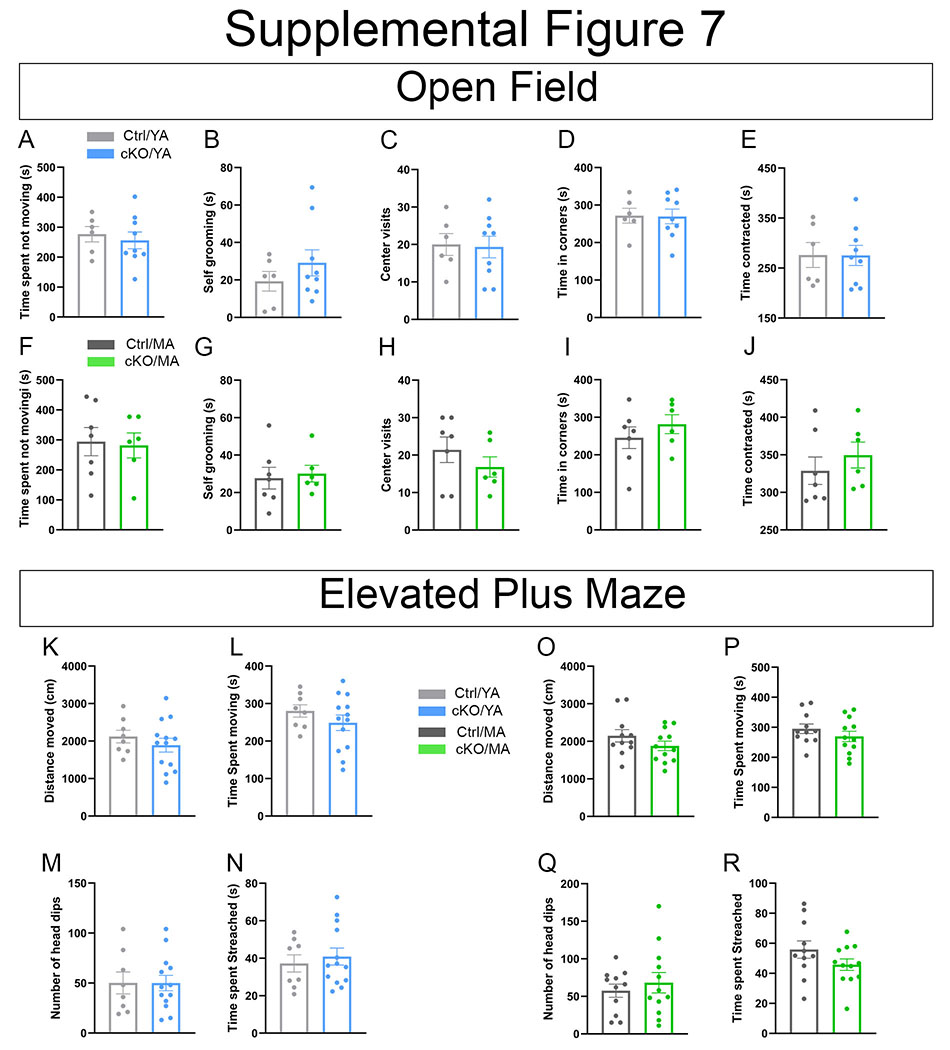

Supplement: Supplementary Figure 7 — Open-field and elevated-plus maze paradigms. Analysis and comparison of TrpV1Cre–/wt;Vmat2flox/flox control (Ctrl) and TrpV1Cre+/wt;Vmat2flox/flox conditional knockout (cKO) mice at young adult (YA; 8 weeks old) and mature adult (MA; 18 weeks old) age in the open field and plus-maze paradigms. Open field test (OFT), Ctrl/YA (N = 6) and cKO/YA (N = 9), Ctrl/MA (N = 7) and cKO/MA (N = 6) data are expressed as mean ± SEM. (A) Time spent not moving YA. (B) Self-grooming YA. (C) Visits to the center YA. (D) Time spent in corners YA. (E) Time spent contracted YA. (F) Time spent not moving MA. (G) Self-grooming MA. (H) Visits to the center MA. (I) Time spent in corners MA. (J) Time spent contracted MA. Elevated plus maze (EPM), Ctrl/YA (N = 8) and cKO/YA (N = 13), Ctrl/MA (N = 11) and cKO/MA (N = 12), data are expressed as mean ± SEM. (K) Distance moved YA. (L) Time spent moving YA. (M) Number of head dips YA. (N) Time spent stretched YA. (O) Distance moved MA. (P) Time spent moving MA. (Q) Number of head dips MA. (R) Time spent stretched MA. [file Image_7.JPEG]

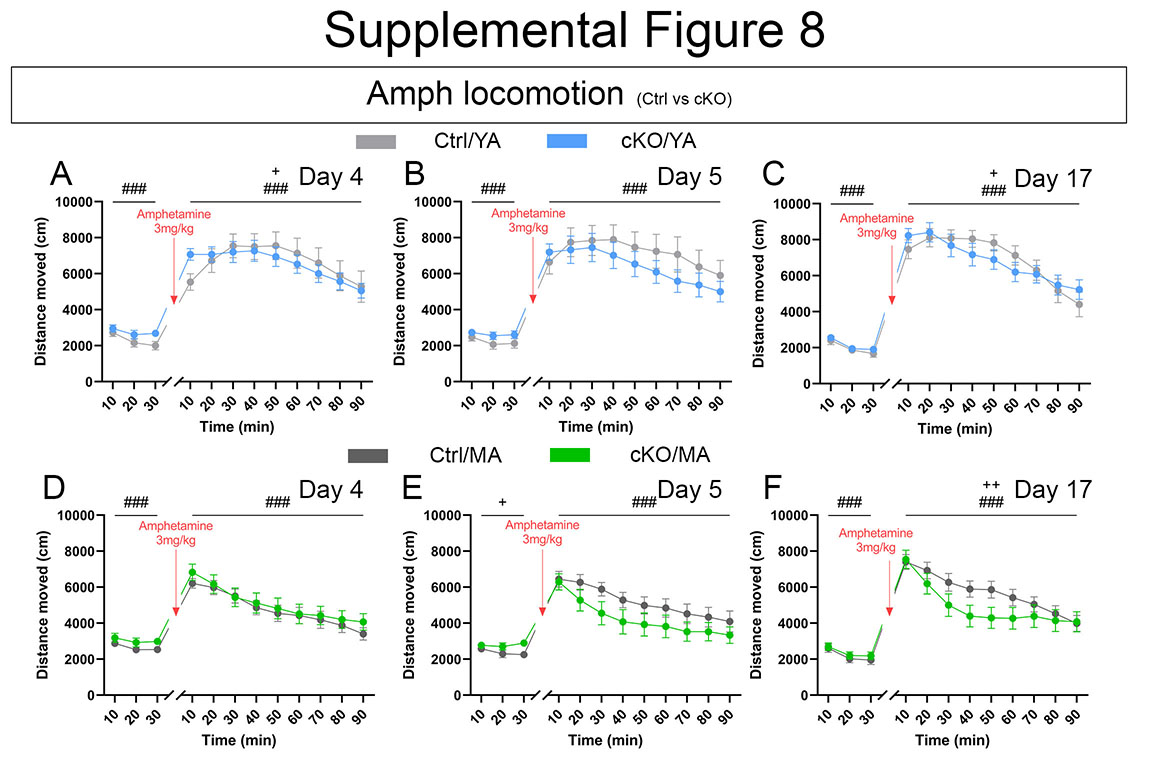

Supplement: Supplementary Figure 8 — Amphetamine-induced locomotion. Analysis and comparison of TrpV1Cre–/wt;Vmat2flox/flox control (Ctrl) and TrpV1Cre+/wt;Vmat2flox/flox conditional knockout (cKO) mice at young adult (YA; 8 weeks old) and mature adult (MA; 18 weeks old) age in the amphetamine sensitization paradigm. Amphetamine-induced locomotion, 90 min following amphetamine injection (YA); Ctrl/YA (N = 22) and cKO/YA mice (N = 22). (A) Amphetamine-induced locomotion on Day 4. Distance moved presented as mean ± SEM for each 10-min period (###p < 0.001, time effect; +p = 0.023, time × genotype interaction). (B) Amphetamine-induced locomotion on Day 5. Distance moved presented as mean ± SEM for each 10-min period (###p < 0.001, time effect). (C) Amphetamine-induced locomotion on Day 17. Distance moved presented as mean ± SEM for each 10-min period (###p < .001, time effect; +p = 0.039, time × genotype interaction). Amphetamine-induced locomotion, 90 min following amphetamine injection (MA); Ctrl/MA (N = 18) and cKO/MA mice (N = 18). (D) Amphetamine-induced locomotion on Day 4. Distance moved presented as mean ± SEM for each 10-min period (###p < 0.001, time effect). (E) Amphetamine-induced locomotion on Day 5. Distance moved presented as mean ± SEM for each 10-min period [###p < 0.001, time effect; +p = 0.02, time × genotype interaction)]. (F) Amphetamine-induced locomotion on Day 17. Distance moved presented as mean ± SEM for each 10-min period (###p < 0.001, time effect; ++p = 0.008, time × genotype interaction). [file Image_8.JPEG]
